# Distinct actions of the fermented beverage kefir on host behaviour, immunity and microbiome gut-brain modules in the mouse

**DOI:** 10.1186/s40168-020-00846-5

**Published:** 2020-05-18

**Authors:** Marcel van de Wouw, Aaron M. Walsh, Fiona Crispie, Lucas van Leuven, Joshua M. Lyte, Marcus Boehme, Gerard Clarke, Timothy G. Dinan, Paul D. Cotter, John F. Cryan

**Affiliations:** 1 APC Microbiome Ireland, University College Cork, Cork, Ireland; 2 Department of Anatomy and Neuroscience, University College Cork, Cork, Ireland; 3 Teagasc Food Research Centre, Moorepark, Fermoy, Cork, Ireland; 4 Department of Psychiatry and Neurobehavioral Science, University College Cork, Cork, Ireland; 5 Microbiology Department, University College Cork, Cork, Ireland

**Keywords:** Microbiota, Kefir, Mouse, Brain, Behaviour, GABA, Immunity, Serotonin, Reward, *Lactobacillus*

## Abstract

**Background:**

Mounting evidence suggests a role for the gut microbiota in modulating brain physiology and behaviour, through bi-directional communication, along the gut-brain axis. As such, the gut microbiota represents a potential therapeutic target for influencing centrally mediated events and host behaviour. It is thus notable that the fermented milk beverage kefir has recently been shown to modulate the composition of the gut microbiota in mice. It is unclear whether kefirs have differential effects on microbiota-gut-brain axis and whether they can modulate host behaviour per se.

**Methods:**

To address this, two distinct kefirs (Fr1 and UK4), or unfermented milk control, were administered to mice that underwent a battery of tests to characterise their behavioural phenotype. In addition, shotgun metagenomic sequencing of ileal, caecal and faecal matter was performed, as was faecal metabolome analysis. Finally, systemic immunity measures and gut serotonin levels were assessed. Statistical analyses were performed by ANOVA followed by Dunnett's post hoc test or Kruskal-Wallis test followed by Mann-Whitney *U* test.

**Results:**

Fr1 ameliorated the stress-induced decrease in serotonergic signalling in the colon and reward-seeking behaviour in the saccharin preference test. On the other hand, UK4 decreased repetitive behaviour and ameliorated stress-induced deficits in reward-seeking behaviour. Furthermore, UK4 increased fear-dependent contextual memory, yet decreased milk gavage-induced improvements in long-term spatial learning. In the peripheral immune system, UK4 increased the prevalence of Treg cells and interleukin 10 levels, whereas Fr1 ameliorated the milk gavage stress-induced elevation in neutrophil levels and CXCL1 levels. Analysis of the gut microbiota revealed that both kefirs significantly changed the composition and functional capacity of the host microbiota, where specific bacterial species were changed in a kefir-dependent manner. Furthermore, both kefirs increased the capacity of the gut microbiota to produce GABA, which was linked to an increased prevalence in *Lactobacillus reuteri*.

**Conclusions:**

Altogether, these data show that kefir can signal through the microbiota-gut-immune-brain axis and modulate host behaviour. In addition, different kefirs may direct the microbiota toward distinct immunological and behavioural modulatory effects. These results indicate that kefir can positively modulate specific aspects of the microbiota-gut-brain axis and support the broadening of the definition of psychobiotic to include kefir fermented foods.

Video abstract.

## Introduction

Mounting evidence suggests that the gastrointestinal microbiota influences host behaviour via bi-directional communication through what has been coined the microbiota-gut-brain axis [17, 18, 20, 29, 50, 60, 71]. Various nutritional interventions have already been demonstrated to influence this axis, with host-indigestible dietary fibres (prebiotics) and live bacterial strains that confer health benefits (probiotics) receiving particular attention [20, 38, 48]. Such interventions that modulate mood through manipulation of the microbiota have been coined psychobiotics [24]. It is becoming apparent that fermented foods may also confer beneficial effects on aspects of mood, as fermented food intake is associated with decreased social anxiety [33] and gestational depression in humans [51]. In addition, a fermented milk product, which was produced using known probiotics, has been demonstrated to modulate brain activity in healthy women [82]. Such findings merit an investigation into the mechanisms by which different fermented foods might affect the microbiota-gut-brain axis.

One such fermented foods are kefir, a traditional fermented milk beverage originating from the Caucasus mountains that is produced by adding a kefir grain to milk. These grains consist of exopolysaccharide matrices harbouring symbiotic microbial communities, including bacteria and yeasts, which together are responsible for fermentation [12]. Notably, the word kefir is derived from the Turkish *keyif*, which translates as “good feeling”. Indeed, numerous health benefits have been ascribed to kefir [62, 75], such as anti-inflammatory effects in animal models [41, 47, 61], reduced obesity symptomatology in high fat diet-induced obese mice [11, 30, 40], and reduced hypertension in spontaneously hypertensive rats [73]. Furthermore, kefir administration has been shown to reduce physical fatigue and improve exercise performance in mice [34]. A recent randomised, controlled trial has even shown that kefir can reduce bloating and improve mood in patients with inflammatory bowel disease [90]. Finally, kefir has been shown to modulate the composition of the gastrointestinal microbiota in rodents [11, 30, 34, 40]. Overall, current evidence indicates that the traditionally fermented milk drink kefir merits investigation to determine its ability to modulate the microbiota-gut-brain axis and affect the mood of the host. It is not clear if kefir’s exert differential influence across the microbiota-gut-brain axis due to their compositional differences [12]. As such, we investigated if two different kefirs could affect the microbiota of ileal, caecal and faecal contents, the faecal metabolome, gastrointestinal function, host adaptive and innate immunity, and behaviour in mice.

## Results

### Kefir microbiota was relatively stable over time

The milk kefir used in this study was generated in a manner to represent traditional kefir production (i.e., repeated fermentation of milk by a kefir grain). Considering that kefir contains a complex microbiota community composed of a variety of strains [12], we determined if this community remained stable over time. Shotgun metagenomics was used to determine the species-level composition of the two kefirs, Fr1 and UK4, at 6 time-points at intervals of 2 weeks throughout the in vivo study. Overall, the populations were generally temporally stable, with both kefirs being dominated by *Lactococcus lactis*, while also consistently containing *L. kefiranofaciens* (Figure S1). Several other species were identified at specific time-points at > 1% relative abundance in both kefirs, such as *Bifidobacterium breve* and *Pseudomonas* species. Notably, *L. kefiranofaciens* was more abundant in kefir Fr1 at some time points.

### The fermented milk drink kefir is well-tolerated

Kefir administration did not affect body weight, body composition, food intake and drinking water intake (Figure S2). In addition, no differences were found in basal body temperature, as detected in the stress-induced hyperthermia test, as well as locomotor activity assessed in the open field test (Figure S2). Overall, this indicates that the fermented milk drink kefir was well-tolerated by mice.

### Kefir did not affect measures of gastrointestinal physiology and motility

Assessment of gastrointestinal motility by carmine red administration showed that kefir did not induce any changes in gastrointestinal propulsion (Figure S3). In line with these findings was the absence of differences in faecal pellet weight and faecal water content (Figure S3). Finally, no differences in caecum weight and colon length were detected at the end of the study (Figure S3). Overall, these data indicate that changes in the gut microbiota are likely independent of host gastrointestinal physiology and motility.

### Kefir modulates repetitive behaviour and reward-seeking behaviour

**Fig. 1:**
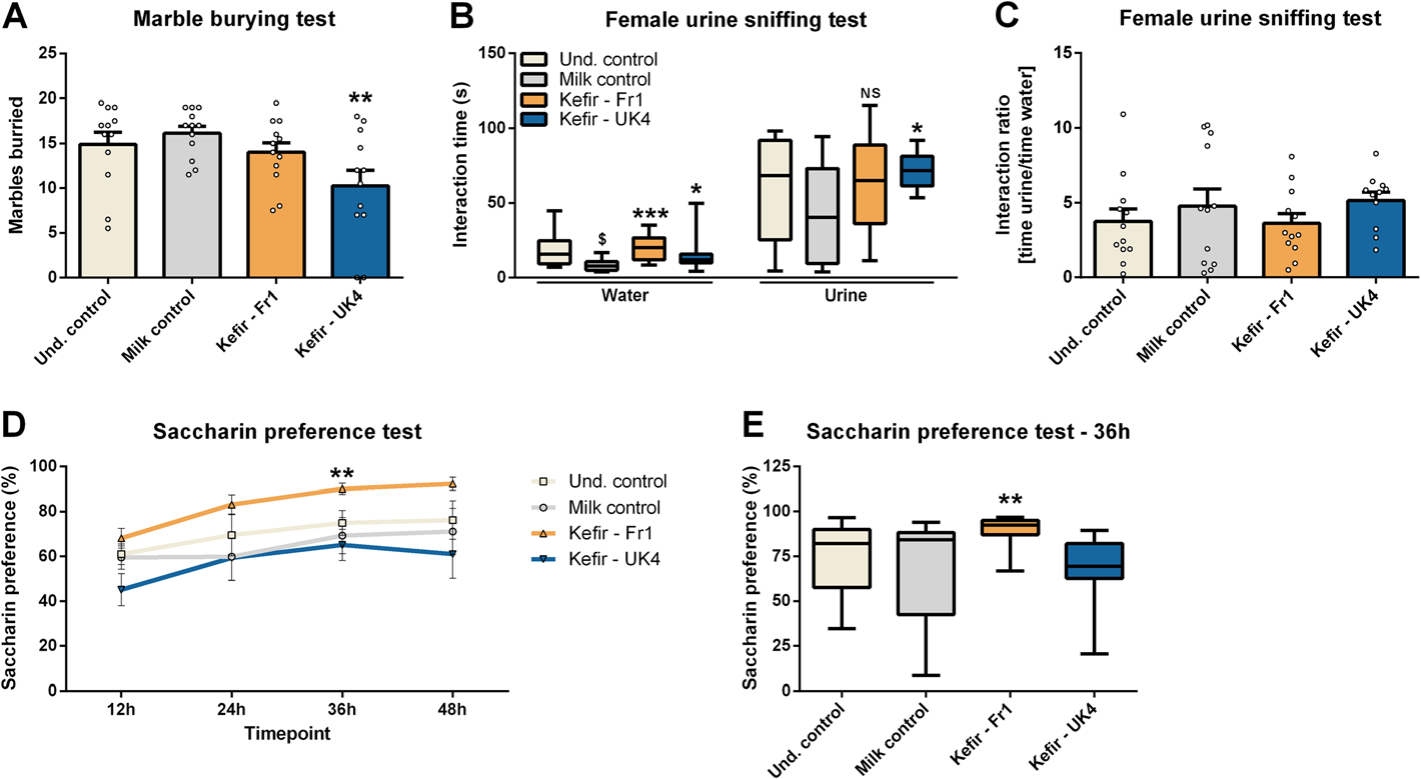
Kefir modulates repetitive behaviour and reward-seeking behaviour. Repetitive/anxiety-like behaviour was assessed using the marble burying test (**a**). Anhedonia and reward-seeking behaviours were investigated using the female urine sniffing test (**b**, **c**) and saccharin preference test (**d**, **e**). The marble burying test was normally distributed and analysed using a one-way ANOVA, followed by a Dunnett’s post hoc test. The female urine sniffing test and saccharin preference test were non-normally distributed and analysed using the Kruskal-Wallis test, followed by the Mann-Whitney test. Significant differences are depicted as *p < 0.05, **p < 0.01 and ***p <0.001; milk control compared to kefir supplementation, ^$^*p* < 0.05; undisturbed control compared to milk control. All data are expressed ‘as mean ± SEM (*n* = 11–12). Dots on each graph represent individual animals

In the marble burying test, we found that administration of UK4 decreased the number of marbles buried indicative of reduced repetitive behaviour (*F*(2,35) = 5.464, *p* = 0.009) (Fig. 1a). No changes were observed in tests assessing anxiety-like behaviours such as the elevated plus maze, open field test and stress-induced hyperthermia test (Figure S4), as well as depressive-like behaviour in the forced swim test and tail-suspension test (Figure S4). Notably, repeated stress of milk gavage increased the corticosterone response to an acute stressor, which remained unaffected by kefir (Figure S4).

In the female urine sniffing test of reward-seeking, mice receiving milk spent less time interacting with the cotton bulb containing water compared to undisturbed mice (*χ*^2^(1) = 6.367, *p* = 0.012), which was ameliorated by both Fr1 and UK4 (*χ*^2^(2) = 13.238, *p* < 0.001) (Fig. 1b). In addition, mice receiving UK4 spent more time interacting with the cotton bulb containing the urine from a female mouse in esterus (*χ*^2^(2) = 6.280, *p* = 0.043) (Fig. 1b), even though no differences were observed in the preference index (Fig. 1c). Finally, Fr1 administration increased saccharin preference in the saccharin preference test (*χ*^2^(2) = 12.826, *p* = 0.002), which is often used as a measure of reward-seeking behaviour (Fig. 1d, e).

### Kefir does not affect sociability

All groups exhibited normal social preference and recognition in the 3-chamber social interaction test, indicating that kefir did not affect sociability (Figure S5).

### Kefir–UK4 modulates contextual learning and memory

**Fig. 2:**
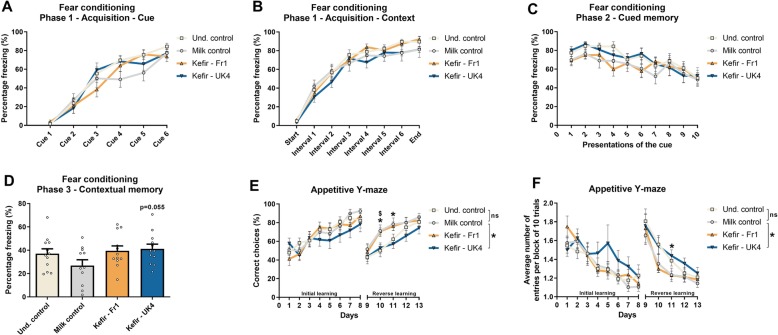
UK4 enhances fear-dependent contextual memory yet decreases long-term spatial learning. Fear-dependent memory and learning were assessed using fear conditioning. At phase 1—acquisition, mice were presented with a tone, followed by a foot shock. Cue-associative learning was assessed by measuring freezing behaviour during the presentation of the tone (**a**), whereas context-associative learning was determined in-between tones (**b**). At phase 2—cued memory, mice received 40 presentations of the same cue (the first 10 are shown), without foot shock, in a different context, in which fear-dependent cued memory was assessed (**c**). At phase 3—contextual memory, mice were exposed to the same context as day one for 5 min and contextual memory was assessed (**d**). Long-term spatial learning was assessed in the appetitive Y-maze, as determined by the percentage of times the mice made the correct choice as the first choice for reaching the goal (food reward) (**e**), as well as the number of average entries it took the mice to reach the goal (**f**). All data were normally distributed and analysed using a repeated measures ANOVA or one-way ANOVA, followed by a Dunnett’s post hoc test. Significant differences are depicted as *p < 0.05; milk control compared to kefir supplementation, ^$^*p* < 0.05; undisturbed control compared to Milk control. All data are expressed as mean ± SEM (*n* = 10–12). Dots on each graph represent individual animals

No differences were observed in the fear conditioning test in phase 1—acquisition, as determined by the time mice spent frozen during the presentation of the cue, as well as in-between the cues (Fig. 2a, b). In addition, no differences were seen during phase 2, when cued-dependent fear memory was assessed (Fig. 2c). However, mice receiving UK4 showed a trend towards increased freezing behaviour in phase 3—contextual memory (*F*(2,34) = 3.181, *p* = 0.055) (Fig. 2d). Conversely, mice receiving UK4 made more errors in the reverse learning phase of the appetitive Y-maze as seen by the percentage correct choices (treatment effect: *F*(2,33) = 3.870, *p* = 0.031) (Fig. 2e), and the amount of entries mice needed to reach the food reward compared to milk control (treatment effect: *F*(2,33) = 3.387, *p* = 0.046) (Fig. 2f). Notably, a similar difference was found on day 10 in the percentage correct choices made between the undisturbed control and Milk control group (*t*(22) = − 2.303, *p* = 0.031), where the mice receiving milk control performed superior to the undisturbed control (Fig. 2e).

### Kefirs differentially impact the peripheral immune system

**Fig. 3:**
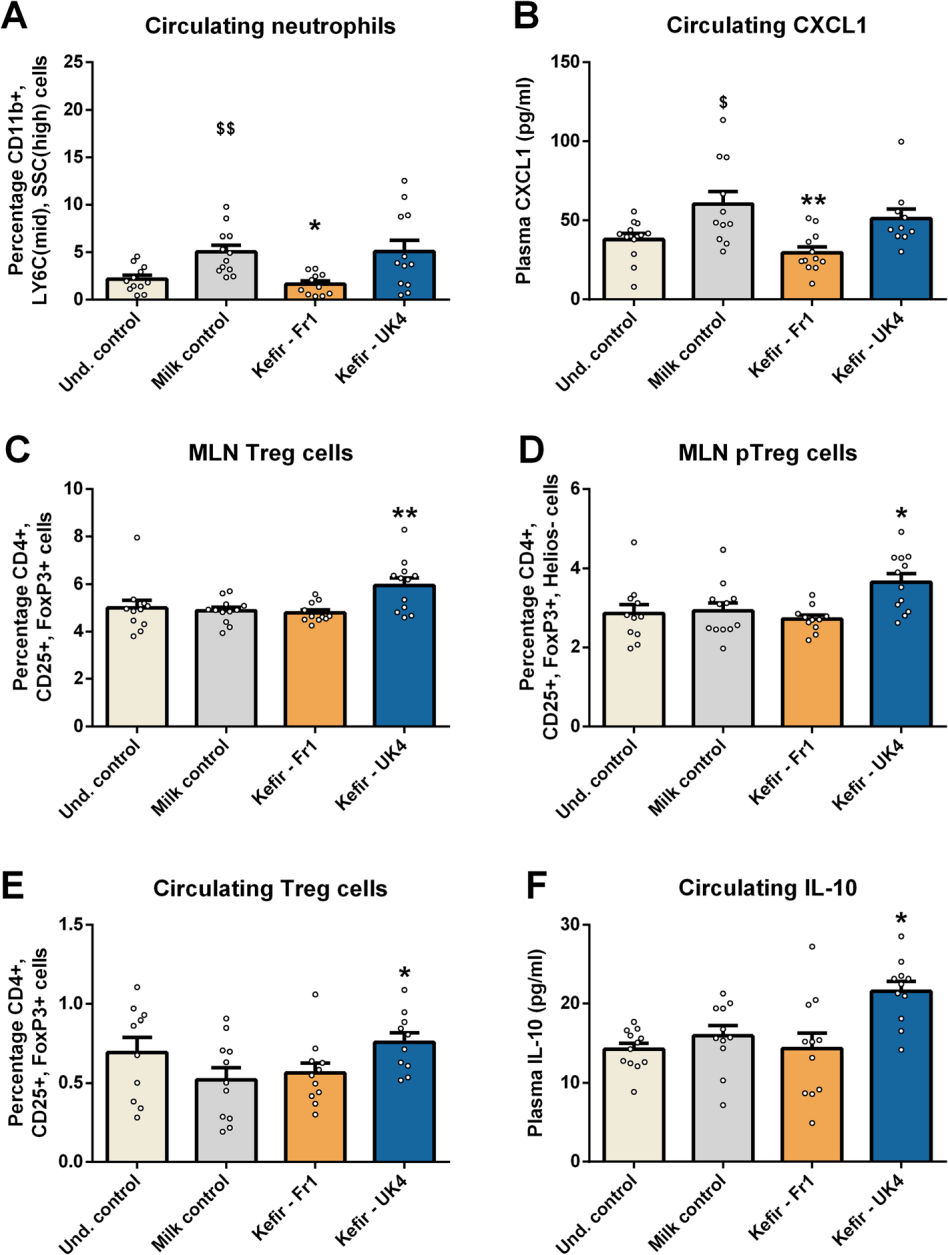
UK4 increases Treg cells levels, while Fr1 decreases neutrophil levels. T regulatory cells (CD4+, CD25+, FoxP3+) were assessed in mesenteric lymph nodes (MLNs) (**a**) and were subsequently analysed for Helios expression (**b**), to investigate their origin as Helios+ cells exclusively originate in the thymus. Blood was also assessed for Treg cell levels (**c**), and plasma interleukin 10 (IL-10) levels (**d**). Similarly, neutrophil (CD11b+, LY6C^mid^, SSC^high^ and CXCL1 levels were also investigated in peripheral blood (**e**, **f**). All data were normally distributed and analysed using a one-way ANOVA, followed by a Dunnett’s post hoc test. Significant differences are depicted as *p < 0.05, **p < 0.01; milk control compared to Kefir supplementation, ^$^*p* < 0.05 and ^$$^*p* < 0.01; undisturbed control compared to milk control. All data are expressed as mean ± SEM (*n* = 11–12). Dots on each graph represent individual animals

There was an increase in circulating LY6C^high^ monocytes in mice receiving milk, compared to undisturbed mice, indicating an activation of the innate immune system [32, 86]. Furthermore, there were increased levels of various inflammatory cytokines in the peripheral circulation (Figure S6). In line with this finding, was an increase in neutrophil levels induced by milk administration (*t*(22) = − 3.583, *p* = 0.002) (Fig. 3a), which was ameliorated by Fr1 administration (*F*(2,34) = 5.412, *p* = 0.009) (Fig. 3a). Similarly, Fr1 ameliorated the increased CXCL1 levels observed in mice chronically stressed by milk gavage (*t*(21) = − 2.589, *p* = 0.017; *F*(2,32) = 7.006, *p* = 0.003) (Fig. 3b), which is one of the major chemoattractants for neutrophils [74].

UK4 increased the prevalence of T regulatory cells (Treg) cells in mesenteric lymph nodes (MLNs) (*F*(2,34) = 8.709, *p* < 0.001) (Fig. 3c), an anti-inflammatory T helper cell subset known to be induced by gut microbial metabolites [80]. These cells did not express the Helios transcription factor (*F*(2,34) = 7.548, *p* = 0.002) (Fig. 3d). This indicated that they were induced in the periphery (pTreg) rather than in the thymus [72], suggesting that gut microbial-derived metabolites could have driven this increase in Treg cells. We subsequently investigated the prevalence of MLN CD103+ dendritic cells, which are known to induce Treg cell differentiation [79]. No corresponding differences were found, as an increased prevalence of MLN CD103+ dendritic cells was observed in the milk and kefir treatment groups, which is in line with the increased levels of inflammatory cytokines in the peripheral circulation (Figure S7). The effects of UK4 also reached the peripheral circulation, where there was an increased prevalence of Treg cells (*F*(2,31) = 3.420, *p* = 0.046) (Fig. 3e). Similarly, we observed increased plasma IL-10 levels (*F*(2,32) = 6.205, *p* = 0.006) (Fig. 3f), one of the primary cytokines secreted by Treg cells [63].

### Kefir–Fr1 selectively increases colonic serotonergic activity

**Fig. 4:**
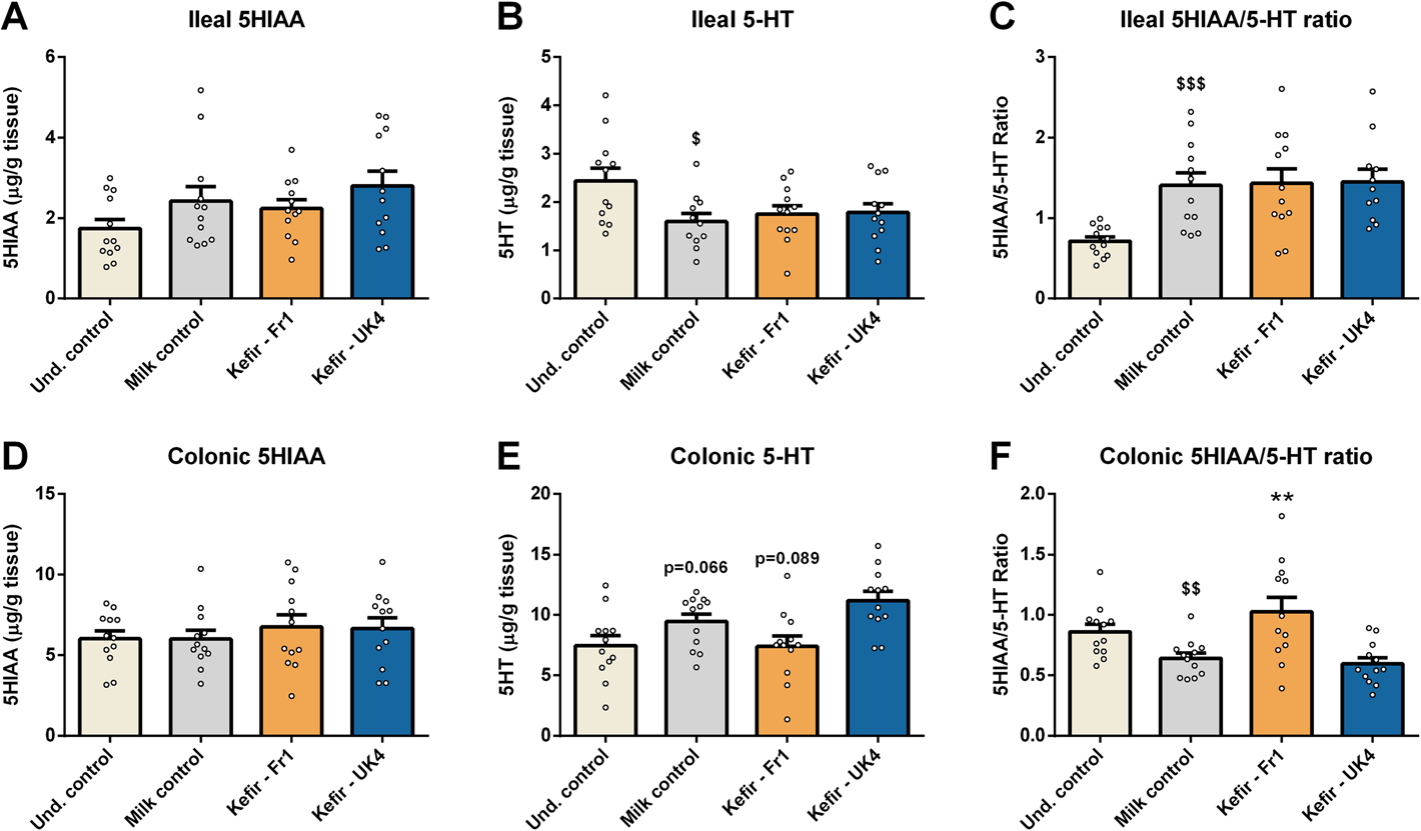
Fr1 modulates serotonergic signalling in the colon, but not ileum. Ileal (**a**–**c**) and colonic (**d**–**f**) tissues were quantified for 5HIAA and serotonin (5-HT) levels using HPLC. The 5HIAA/5-HT ratio was subsequently calculated. All data were normally distributed and analysed using a one-way ANOVA, followed by a Dunnett’s post hoc test. Significant differences are depicted as: **p < 0.01; milk control compared to kefir supplementation, ^$^*p* < 0.05, ^$$^*p* < 0.01 and ^$$$^*p* < 0.001; Undisturbed control compared to milk control. All data are expressed as mean ± SEM (*n* = 11–12). Dots on each graph represent individual animals

Serotonergic signalling is well-known to play a key-role in microbiota-host cross-talk [55, 78], which is why we quantified gut serotonin (5-HT) levels. We found that mice receiving milk showed decreased ileal 5-HT levels compared to undisturbed mice (*t*(21) = 2.650, *p* = 0.015) (Fig. 4b). This resulted in an increased 5HIAA/5-HT ratio (*t*(22) = 2.650, *p* < 0.001) (Fig. 4c), indicating an increased serotonin turnover and serotonergic activity. The opposite was observed in the colon, where the milk induced a trend towards increased 5-HT levels (*t*(22) = − 1.937, *p* = 0.066) (Fig. 4e), whilst decreasing the 5HIAA/5-HT ratio (*t*(22) = 2.907, *p* = 0.008) (Fig. 4f). This phenotype in the colon, but not ileum, was ameliorated by Fr1 (for 5-HT; *F*(2,35) = 6.387, *p* = 0.005, for 5HIAA/5-HT ratio; *F*(2,35) 9.026, *p* < 0.001) (Fig. 4e, f).

### Both kefirs affect gut microbiota composition, at both the species- and strain-levels

**Fig. 5:**
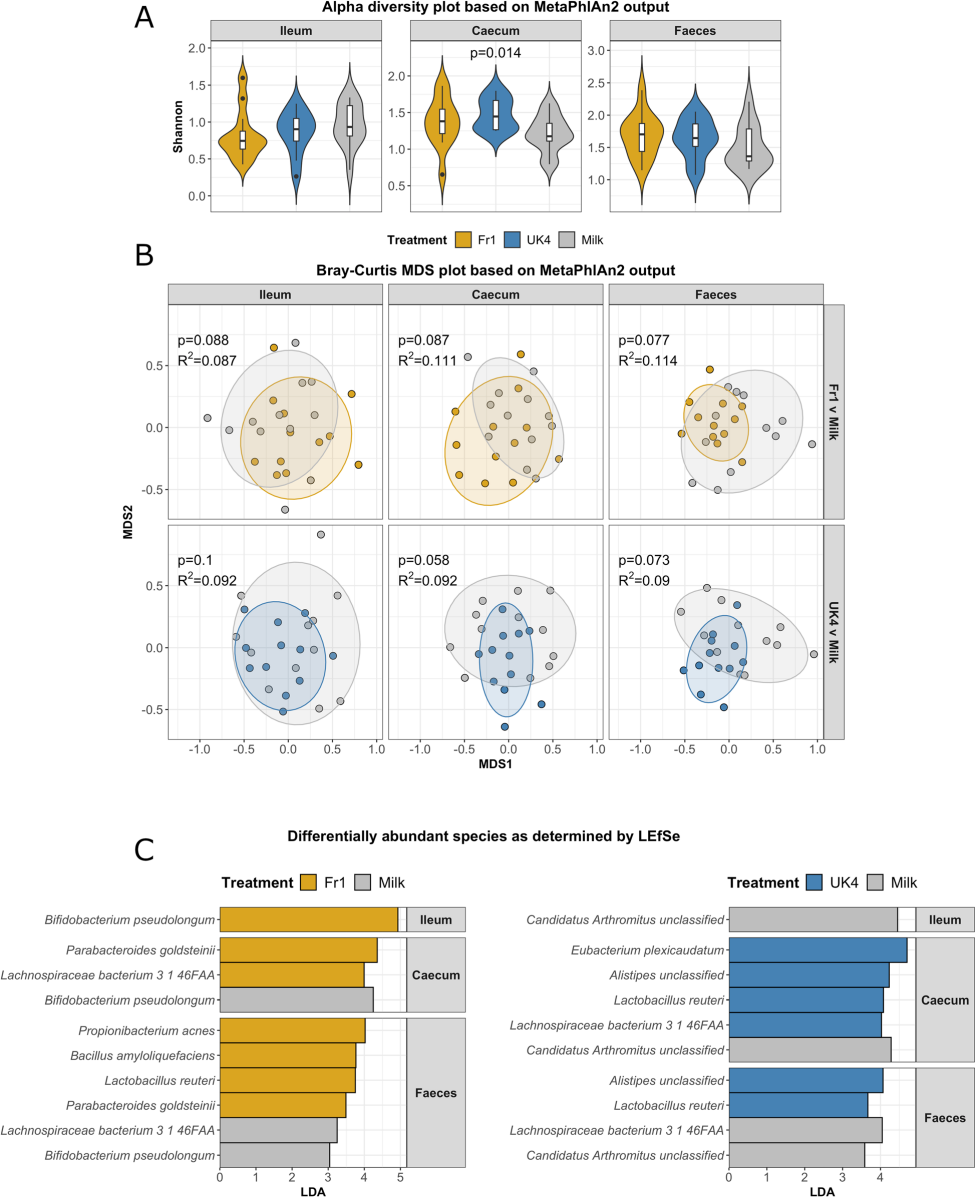
Kefir modulates the composition of the gastrointestinal microbiota. Alpha diversity (Shannon) of the ileal, caecal and faecal microbiota was compared between mice receiving kefir (Fr1 or UK4) and mice receiving milk control using violin plots (**a**). Beta diversity was assessed by PERMANOVA to investigate the dissimilarity in the gut microbial composition, which were depicted using MDS plots. Dots represent individual animals (*n* = 12) (**b**). Differentially abundant taxa were determined using LEfSe (**c**)

We subsequently investigated if kefir administration could affect the composition of the ileal, caecal and faecal microbiota. Alpha diversity (Shannon) was not significantly altered by kefir Fr1 administration (Fr1; ileum *p* = 0.11; caecum *p* = 0.19; faeces *p* = 0.16), whereas kefir UK4 increased alpha diversity in the caecum (*p* = 0.017), but not ileum and faeces (*p* = 0.44; *p* = 0.24) (Fig. 5a). Analysis of beta diversity revealed a trend towards significant separation induced by the administration of Fr1 (ileum *p* = 0.088, *R*^2^ = 0.111; caecum *p* = 0.087, *R*^2^ = 0.087; faeces *p* = 0.077, *R*^2^ = 0.114) and UK4 (ileum *p* = 0.058, *R*^2^ = 0.092; caecum *p* = 0.1, *R*^2^ = 0.092; faeces *p* = 0.073, *R*^2^ = 0.09) (Fig. 5b). It is notable that, the kefir treatment overall did influence beta diversity (*p* value ranged from 0.05 < *p* ≤ 0.10 for all regions). Notably, no significant differences were found between the administration of Fr1 and UK4 in alpha diversity (ileum *p* = 0.37; caecum *p* = 0.34; faeces *p* = 0.8) and beta diversity (ileum *p* = 0.316, *R*^2^ = 0.05; caecum *p* = 0.14, *R*^2^ = 0.07; faeces *p* = 0.2, *R*^2^ = 0.062).

A total of 15 bacterial species were identified as being differentially abundant between at least one pair of groups in at least one region of the gut (Fig. 5c). Both kefirs increase the abundance in or more region in the gut of *Lactobacillus reuteri* (Fr1, caecum LDA = 4.36, UK4, caecum LDA = 4.02, UK4, faeces LDA = 4.07), *Eubacterium plexicaudatum* (Fr1, faeces LDA = 3.77, UK4, caecum: LDA = 4.22, UK4, faeces LDA = 3.67)*, Bifidobacterium pseudolongum* (Fr1, ileum LDA = 4.93, UK4, caecum LDA = 4.7). Both kefirs induced a decrease in the prevalence of *Lachnospiraceae bacterium* 3_1_46FAA (Fr1, caecum LDA = 4.25, UK4 caecum: LDA = 4.28), *Propionibacterium acnes* (Fr1, faeces LDA = 3.25, UK4, faeces, LDA = 4.04), and *Bacillus amyloliquefaciens* (Fr1, faeces LDA = 3.04, UK4, faeces LDA = 3.58). Only Fr1 increased the prevalence of *Parabacteroides goldsteinii* (caecum LDA = 3.99), *Bacteroides intestinalis* (faeces: LDA = 3.49), *Anaerotruncus* unclassified (faeces: LDA = 3.75), and *Parabacteroides goldsteinii* (faeces LDA = 4.02). Conversely, only UK4 increased the prevalence of *Alistipes* unclassified (caecum: LDA = 4.45) and decreased *CandidatusArthromitus* unclassified (ileum LDA = 4.45).

**Fig. 6:**
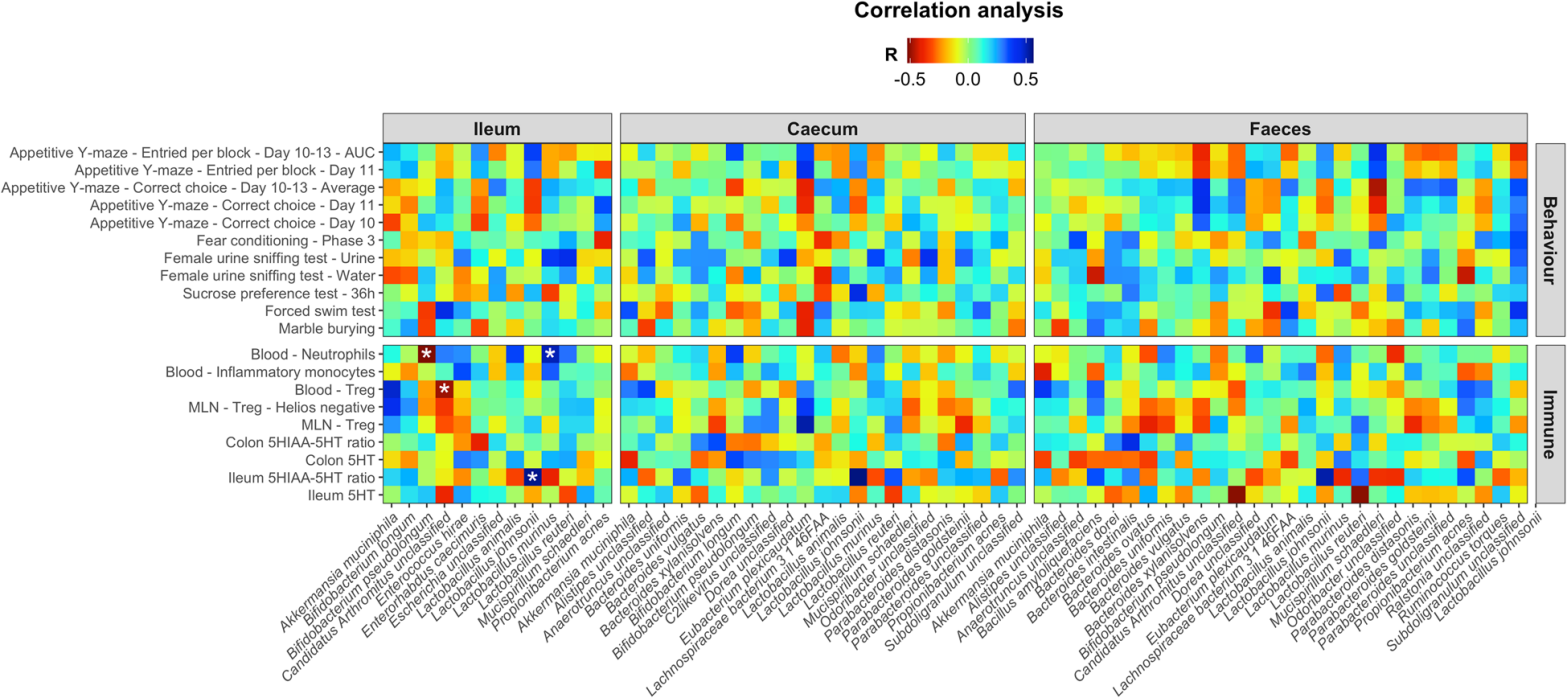
Bacterial species in the gastrointestinal microbiota correlate with changes in host immunity and measures of gut serotonin. The prevalence of bacterial species in the ileum, caecum and faeces were correlated with significantly altered changes in host behaviour and immunity using HAllA

We subsequently correlated significantly altered behavioural and immunological parameters with bacterial species present throughout the gastrointestinal microbiota (Fig. 6). Most notable was the correlation between ileal *C. Arthromitus* unclassified abundances and circulating Treg cell levels (*p* = 0.004, *R* = − 0.49), and ileal *B. pseudolongum* abundances and circulating neutrophil levels (*p* = 0.001, *R* = − 0.52).

PanPhlAn was used alongside StrainPhlAn to characterise differentially abundant species to the strain-level. Both tools indicated that the detected *B. pseudolongum* strain was closely related to *B. pseudolongum* UMB-MBP-01 (Figure S8). Similarly, PanPhlAn indicated that the detected *L. reuteri* strain was closely related to *L. reuteri* TD1. No other differentially abundant species could be characterised to the strain-level. Finally, neither PanPhlAn nor StrainPhlAn identified any kefir-derived strains in the gut microbiota of mice receiving kefir, establishing that the microbiota of the administered kefir did not colonise to high levels.

### Kefir induces shifts in the functional potential of the gut microbiome

**Fig. 7:**
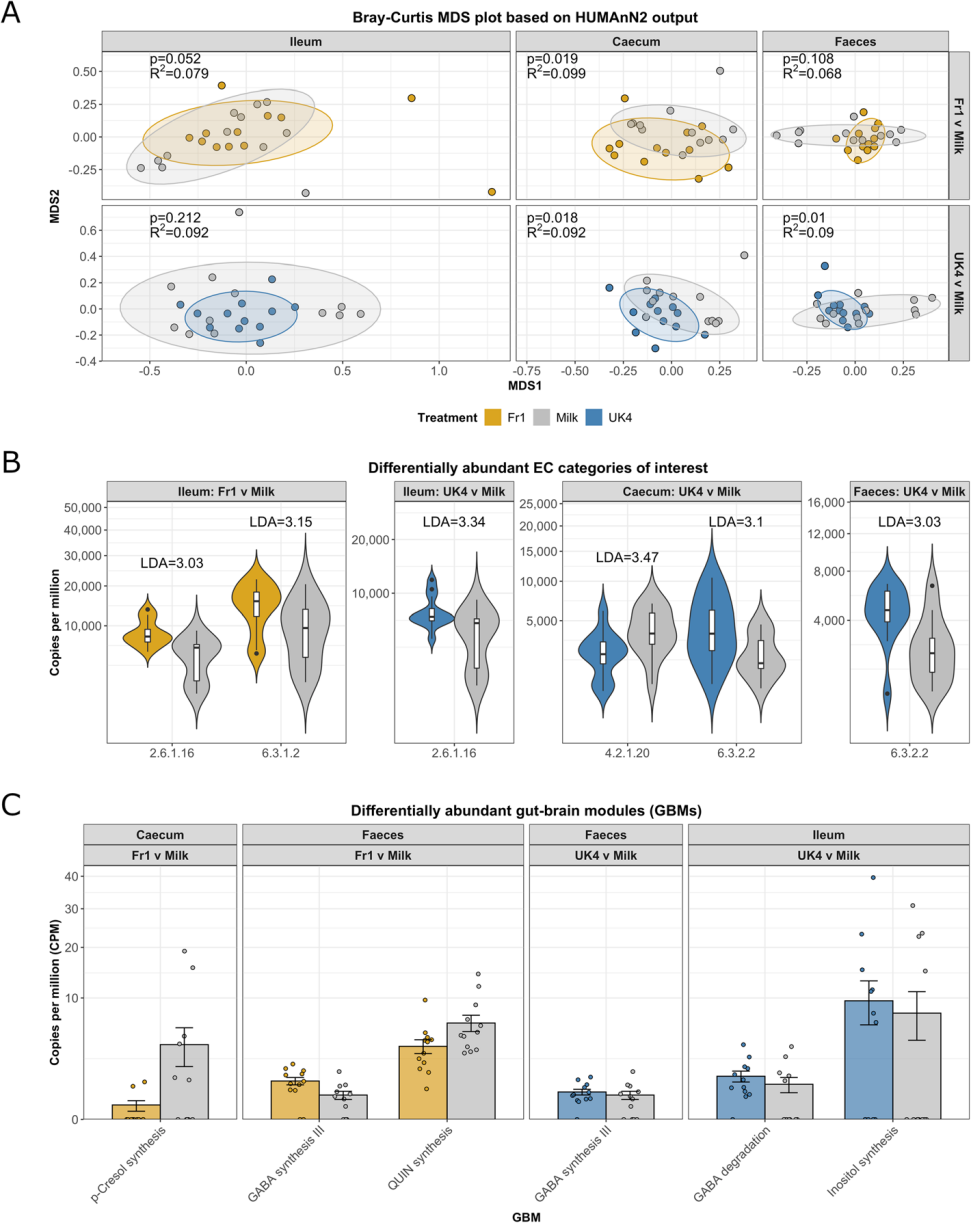
Kefir modulates the functional capacity of the gastrointestinal microbiota. Beta diversity was assessed by PERMANOVA to investigate the dissimilarity in the functional capacity of the gut microbiota, which were depicted using MDS plots. Dots represent individual animals (*n* = 12) (**a**). Differential abundances were assessed of enzyme categories (EC) and depicted as violin plots (**b**). Gut-brain modules (GBMs) were additionally assessed for their differential abundance (**c**)

We subsequently investigated if kefir administration could affect the functional potential of the microbiome in mice. Fr1 induced a significant functional separation in the microbiome in the ileum (*p* = 0.052, *R*^2^ = 0.099) and the caecum (*p* = 0.019, *R*^2^ = 0.079), but not in the faeces (*p* = 0.108, *R*^2^ = 0.068) (Fig. 7a). Similarly, UK4 induced a significant functional separation in the caecum (*p* = 0.018, *R*^2^ = 0.092) and the faeces (*p* = 0.010, *R*^2^ = 0.09), but not in the ileum (*p* = 0.212, *R*^2^ = 0.092) (Fig. 7a). No significant functional separations were identified between any other pair of groups in any other regions. The faecal metabolome was assessed using GC-MS from a subset of animals from each group (*n* = 6) to validate any changes observed in the predicted functional potential of the gut microbiome (Table 3). Analysis of the beta diversity of the faecal metabolome revealed a predominant effect of kefir Fr1 on the measured metabolites (*p* = 0.045, *R*^2^ = 0.196). Notably, no significant differences in the concentrations of any compound between any pair of groups were identified following p-value adjustment, which included short-chain fatty acid (SCFA) levels (Figure S9).

A total of 59 level-4 enzyme commission (EC) categories were differentially abundant between at least one pair of groups in at least one region of the gut. Notably, there were significant differences in several EC categories involved in the production of neuroactives (Fig. 7b). Specifically, ileal glutamine--fructose-6-phosphate transaminase (isomerising) (EC 2.6.1.16) levels, which produces glutamate, was elevated by the administration of both Fr1 (*p* = 0.002) and UK4 (*p* = 0.021). In addition, glutamate--ammonia ligase (EC 6.3.1.2), which produces glutamine, was higher in the ileum of mice receiving Fr1 (*p* = 0.024). UK4 increased the prevalence of predicted caecal and faecal glutamate–cysteine ligase (EC 6.3.2.2) levels (*p* = 0.038; *p* = 0.011 respectively), while caecal tryptophan synthase (EC 4.2.1.20) was decreased (*p* = 0.028).

### Kefir increases the prevalence of a *Lactobacillus reuteri* strain with the potential to produce GABA

Subsequently, changes in the microbiome were explored in the context of the gut-brain axis by examining the abundances of gut-brain modules (GBMs) (Fig. 7c), which are groups of KEGG Orthogroups (KOs) that are associated with the production of neuroactive compounds [85]. In mice receiving Fr1, caecal “p-Cresol biosynthesis” was decreased, while “Quinolinic acid synthesis” was decreased in the faeces (LDA = 4.43) compared to milk control. In mice receiving UK4, there was an increase in ileal “inositol synthesis” (LDA = 4.77) and “GABA degradation” (LDA=4.70). Finally, the GBM “GABA synthesis III” was significantly higher in mice receiving either kefir (Fr1 LDA = 4.39; UK4 LDA = 4.21) in the faeces. These increases in “GABA synthesis III” were attributed to *L. reuteri*, which showed a significantly higher prevalence of this GBM compared to milk controls (Fr1 LDA = 4.20, UK4 LDA = 4.11). Similarly, the GBM “S-adenosylmethionine synthesis” from *P. goldsteinii* was significantly higher in the faeces of mice receiving Fr1 (LDA = 4.21) and UK4 (LDA = 4.08). Furthermore, the GBM “GABA degradation” from *L. reuteri* was significantly higher in the ileum of mice receiving UK4 (LDA = 4.16). While “glutamate synthesis I” from *B. pseudolongum* was higher in the faeces of Fr1 mice (LDA = 4.64).

To corroborate the results from HUMAnN2, PanPhlAn gene-family matrices were examined to identify genes associated with the production of neurotransmitters in the detected strains. The detected *B. pseudolongum* strain encoded two enzymes involved in the production of glutamate: glutamate synthase and glutamine–fructose-6-phosphate transaminase (isomerising). Furthermore, this strain also encoded a glutamate/GABA antiporter, which may be involved in exporting glutamate from the cell. Similarly, the detected *L. reuteri* strain was also found to encode two enzymes associated with the production of glutamate from glutamine: glutaminase and glutamine–fructose-6-phosphate transaminase (isomerising). Importantly, this strain encoded glutamate decarboxylase, which produces GABA by the decarboxylation of glutamate, along with a glutamate/GABA antiporter.

**Fig. 8:**
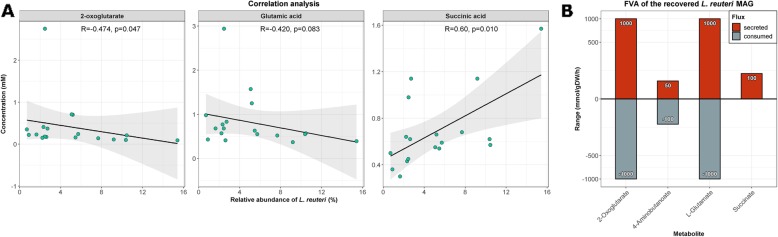
The kefir-induced increased *Lactobacillus reuteri* strain has the potential to produce GABA. Spearman rank correlations between *Lactobacillus reuteri* abundances and levels of faecal 2-oxoglutarate, glutamate and succinate were performed (**a**). Faecal metabolite levels were quantified using chromatography–mass spectrometry (GC-MS). Flux variability analysis was used to assess whether *L. reuteri* can influence 2-oxoglutarate, glutamate and succinate levels (**b**)

In line with the predicted capacity of *L. reuteri* to produce GABA, we observed a negative correlation between *L. reuteri* and faecal levels of 2-oxoglutarate (*R* = − 0.474, *p* = 0.047) and glutamate (*R* = − 0.420, *p* = 0.083), as detected by GC-MS (Fig. 8a). Furthermore, there was a trend towards a positive correlation between *L. reuteri* and succinate levels (*R* = 0.60, *p* = 0.100) (Fig. 8a). Furthermore, faecal succinate levels were increased in mice receiving kefir (Fr1 unadjusted *p* = 0.037; UK4 unadjusted *p* = 0.030). The pattern of correlation suggests that *L. reuteri* was potentially converting 2-oxoglutarate to glutamate, which was subsequently converted into GABA, resulting in the production of succinate as a by-product. Subsequently, we employed metabolic modelling using FVA of *L. reuteri* to explore this hypothesis in depth. The results indicated that *L. reuteri* had the ability to consume 2-oxoglutarate and glutamate, while it also could secrete GABA and succinate (Figure 8b).

## Discussion

In the present study, we demonstrate that two different traditionally fermented kefirs differentially affect host behaviour and immunity. For instance, Kefir Fr1 increased reward-seeking behaviour and ameliorated stress-induced increases in circulating neutrophil and CXCL1 levels. Furthermore, UK4 decreased repetitive behaviour, increased circulating Treg cells and IL-10 levels, and ameliorated deficits in reward-seeking behaviour induced by chronic oral gavage stress. In addition, UK4 increased fear-dependent contextual memory, yet decreased milk gavage-induced improvements in long-term spatial learning. Both kefirs modulated the composition and functional capacity of the microbiota, which was associated with an increased capacity to produce GABA. This function was linked to an increased prevalence of *L. reuteri*.

We observed some changes in the gut microbiota that were specific to kefir Fr1, such as an increase in *P. goldsteinii*, *B. intestinalis*, *Anaerotruncus* unclassified and *P. goldsteinii*. Analysis of GBMs revealed that Fr1 decreased the GBM “p-Cresol biosynthesis” and “inositol synthesis”, while UK4 increased “inositol synthesis”. The increase in *P. goldsteinii* abundances were linked to an increase in the GBM “S-adenosylmethionine synthesis”. S-adenosylmethionine supplementation has been studied in numerous randomised, controlled trials involving depressed adults [36, 70]. Notably, one of the key features of depression is decreased reward-seeking behaviour (i.e. anhedonia) and that Fr1 increased reward-seeking behaviour in the saccharin preference test. As such, the increase in predicted S-adenosylmethionine synthesis could indicate increased levels of S-adenosylmethionine, which in turn might have contributed to the Fr1-induced increase in reward-seeking behaviour. It is important to note, however, that no changes were observed in other measures of depressive-like behaviour. Our results also reveal that Fr1 ameliorated stress-induced deficits in colonic serotonergic signalling, even though it is important to note that no corresponding serotonin-related changes were observed in the gut microbiome and metabolome and that the determined effects were weak. Furthermore, Fr1 increased circulating neutrophil and CXCL1 levels. Notably, p-Cresol, of which the predicted biosynthesis was decreased by Fr1, alters neutrophil function in dogs [10]. In addition, Fr1 increased *B. pseudolongum* abundances in the ileum, whereas UK4 increased *B. pseudolongum* abundances in the caecum. In tandem, ileal *B. pseudolongum* abundances correlated with circulating neutrophil levels, indicating that *B. pseudolongum* abundances in the ileum specifically, might contribute to the decrease in neutrophil levels observed in mice receiving Fr1.

Some of the other changes in the composition of the microbiota were specific to UK4, such as an increased prevalence of *Alistipes* unclassified and decreased *C. Arthromitus* unclassified. In tandem, UK4 decreased repetitive behaviour and modestly increased reward-seeking behaviour in the female urine sniffing test, also often used as a measure of depression-related anhedonia. It is important to note, however, that no changes were observed in other measures of depressive-like behaviour. UK4 also decreased milk gavage-induced improvements in long-term spatial learning, yet increased fear-dependent contextual memory, indicating that not all behavioural effects induced by kefir are positive. Finally, UK4 increased circulating Treg cells and IL-10 levels. In tandem, ileal *C. Arthromitus* unclassified abundances correlated with circulating Treg cell levels, indicating that this bacterial species is likely contributing to the increase in Treg cell levels induced by UK4. Notably, supplementation of *L. kefiri* CIDCA 8348 results in increased gene expression of IL-10 in the ileum and MLNs in mice [15]. In addition, specific bacterial species that were increased by kefir administration, such as *B. pseudolongum* and *L. reuteri*, have previously been associated with increased levels of the anti-inflammatory cytokine IL-10 in mice and Treg cells in mice and humans, respectively [53, 65].

Analysis of the gut microbiota revealed that UK4 increased alpha diversity in the caecum, but no other changes in alpha diversity were observed in any other region or comparison. Analysis of beta diversity revealed a trend towards significant separation induced by both Fr1 and UK4, which resulted in a significant separation induced by kefir overall. Changes in the overall composition of the gut microbiota induced by kefir, or kefir-associated bacterial strains have been reported previously [15, 30, 34, 40, 90]. Notably, none of the bacterial strains present in the kefir microbiota were detected in the gut microbiota of mice receiving kefir, indicating that the kefir microbiota did not colonise to high levels. This has parallels with the fact that probiotics most frequently do not colonise the gut [21]. Our analysis additionally revealed that both kefirs increased the prevalence of *L. reuteri*, *E. plexicaudatum*, *B. pseudolongum*, while decreasing of *L. bacterium* 3_1_46FAA, *P. acnes*, and *B. amyloliquefaciens*. Notably, *L. reuteri* is a bacterial strain that has been ascribed numerous beneficial effects on host immunity and metabolism [52, 53]. Notably, previous reports have also demonstrated an increased prevalence of *Lactobacillus* species in response to kefir supplementation [40], as well as *L. kefiri* CIDCA 8348 administration, a species frequently found in kefir [15].

Our data also reveals that both kefirs significantly modulated the functional capacity of the gut microbiota and altered the levels of GBMs, which was related to the prevalence of specific bacterial strains. Importantly, kefir-induced increases in *L. reuteri* levels were linked to an increased capacity to produce GABA. Indeed, *Lactobacillus* strains have previously been reported to produce GABA [6, 7, 46]. Our data additionally reveals that *L. reuteri* encodes enzymes and antiporters associated with GABA production. Furthermore, levels of faecal metabolites associated with GABA synthesis (i.e. 2-oxoglutarate, glutamate and succinate) correlate with *L. reuteri* abundances. GABA is the primary inhibitory neurotransmitter in the brain and central GABA levels have been linked to anxiety and depression [7], indicating that enhancing GABA production in the gut might be associated with anxiety- and depressive-like behaviour. Indeed, two GABA-producing *Lactobacillus* strains have recently been shown to reduce depressive-like behaviour in high fat diet-induced obese mice [58]. Various *Lactobacillus* strains, or *Lactobacillus*-containing supplements, have been shown to reduce depressive-like behaviour in rodents [1, 13, 23, 44, 45] and improve measures of depression in humans [3, 5, 39, 76]. Furthermore, abundances of faecal GABA-producers, such as *Bacteroides*, correlate negatively with brain signatures associated with depression [77]. Notably, one of the key features of depression is decreased reward-seeking behaviour (i.e. anhedonia) and that both kefirs increased reward-seeking behaviour. It is, however, important to note that it is still unclear whether gut-derived GABA can cross the blood-brain barrier and whether increasing gut-derived GABA levels can improve mood [9].

## Conclusion

These data demonstrate that kefir can modulate specific aspects of the microbiota-gut-brain axis in mice, supporting the recent broadening of the definition of psychobiotics to include fermented foods, such as the fermented milk drink kefir [64]. In addition, both kefirs differentially affected repetitive behaviour and reward-associated behaviour. In line with these findings, kefirs differentially impacted systemic immunity and colonic serotonergic signalling. Furthermore, kefir influenced specific gut microbial functional capacities, including the biosynthesis of various neuroactives such as GABA. These changes in the gut microbiota function and peripheral immunity might contribute to the kefir-induced behavioural phenotype, even though more research is warranted to validate whether these specific microbiota-gut-brain axis pathways are involved at all. Moreover, studies on the validation of kefir as a dietary intervention to improve mood in humans are now warranted.

## Methodology

### Animals

This study used male C57BL/6j mice (8 weeks of age on arrival; Envigo, UK; *n* = 12/group, *n* = 48 in total). Treatment groups were divided into 1) (cow’s) Milk control, 2) Kefir gavage–Fr1, 3) Kefir gavage UK4, 4) undisturbed control. The last group was added to control for the fact that chronic oral gavage or milk administration could affect behaviour or physiology [87]. Food and drinking water were provided *ad libitum* throughout the study. Animals were housed in groups of 4. The holding room had a temperature of 21 ± 1 °C and humidity of 55 ± 10% with a 12-h light/dark cycle (lights on at 7:00 am). Bodyweight was monitored on a weekly basis. Experiments were conducted under the project authorisation license B100/3774 in accordance with the European Directive 86/609/EEC and the Recommendation 2007/526/65/EC and approved by the Animal Experimentation Ethics Committee of University College Cork. All efforts were made to reduce the number of animals used and to minimise the suffering of these animals.

### Experimental timeline and behavioural testing

**Fig. 9:**
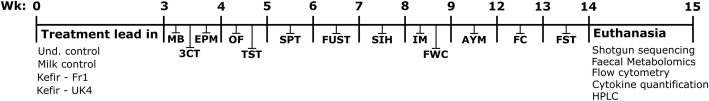
Experimental design. Animals received three weeks of treatment lead in, where animals only received kefir or milk supplementation, after which they were assessed for their behavioural phenotype. Treatment groups consisted of (1) no gavage control, (2) milk gavage control, (3) kefir gavage–Fr1, and (4) kefir gavage–UK4 (*n* = 12/group). The order of behavioural tests was as following: week 4: marble burying test (MB), 3-chamber social interaction test (3CT) and elevated plus maze (EPM); week 5: open-field test (OF) and tail suspension test (TST); week 6: saccharin preference test (SPT); week 7: female urine sniffing test (FUST); week 8: stress-induced hyperthermia test (SIH); week 9: intestinal motility test (IM) and faecal water content assessment (FWC): week 9–12: appetitive Y-maze; week 13: fear conditioning; week 14: forced swim test; week 15: euthanasia. Postmortem, ileal, caecal and faecal microbiota composition and function were investigated by shotgun sequencing and faecal metabolomics. Host immunity was assessed using flow cytometry and by quantification of cytokines. Finally, ileum and colonic serotonergic levels were quantified by high-performance liquid chromatography (HPLC)

Animals were habituated for 1 week prior to the onset of daily kefir administration by oral gavage. After 3 weeks of treatment, animals were assessed for their behavioural phenotype using various tests, which were formed in order of least stressful to most stressful to reduce the likelihood of prior behavioural tests influencing subsequent ones (Fig. 9). In addition, there was a minimum of 36 h between tests. The order of testing was as follows: (1) marble burying test, (2) 3-chamber social interaction test, (3) elevated plus maze, (4) open-field test, (5) tail-suspension test, (6) saccharin preference test, (7) female urine sniffing test, (8) stress-induced hyperthermia test, (9) intestinal motility test, (10) assessment of faecal water content and weight, (11) appetitive Y-maze, (12) fear conditioning, (13) forced swim test*.* At the end of the study, body composition (i.e. percentage lean, fat and fluid mass) was assessed (Minispec mq 7.5), after which animals were immediately sacrificed by decapitation.

### Kefir culturing and administration

Kefir grains were cultured in Irish whole full fat cow’s milk (2% w/v) at 25 °C and milk were renewed every 24 h using a sterile Buchner funnel and sterile Duran bottle, as previously described [25, 88]. Grains were rinsed with deionised water prior to the renewal of milk. The fermented milk (i.e. kefir) collected after 24-h culturing, or unfermented milk control, were administered to the mice within 1 h by oral gavage (0.2 mL). The same milk was used for the unfermented milk control and was similar to the kefir, also incubated for 24 h at 25 °C. Daily kefir administration was performed after the behavioural test, if one was performed that day, between 4.00 and 7.00 pm. To analyse the kefir microbiota over time, aliquots from the kefir administered to the mice were taken on a weekly basis and stored at –80 °C for subsequent analysis.

### Marble burying test

Mice were tested for repetitive behaviour with the marble burying test [81], which was conducted as previously described [14]. Animals were individually placed in a novel Plexiglas cage (35 × 28 × 18.5 cm, L × W × H), which was filled with sawdust (5 cm) and had 20 equally spread marbles placed on top (5 × 4 rows). After mice had spent 30 min in the cage, the number of buried marbles was counted by two researchers and averaged. A buried marble was defined as 2/3 of the marble not being visible anymore. Sawdust was renewed, and marbles cleaned with 70% ethanol in-between animals.

### 3-chamber social interaction test

The three-chamber sociability test was used to assess social preference and recognition and was conducted as previously described [22]. The testing apparatus was a three-chambered, rectangular box. The dividing walls between each chamber (20 × 40 × 22 cm, L × W × H) had small circular openings (5 cm diameter), allowing for access to all chambers. The two outer chambers contained wire cup-like cages (10 cm bottom diameter, 13 cm height), allowing for auditory, olfactory and visual, but not physical contact. The test consisted of three 10-min phases: (1) habituation, (2) social preference, (3) social recognition. In the first phase (habituation), mice were allowed to explore the entire box with both wire cup-like cages left empty to allow for habituation to the novel environment. In the second phase (social preference), one wire cup-like cage contained a novel, age-matched, conspecific, male mouse, whereas the other cage contained an object (rubber duckie). In the third phase (social recognition), the mouse of the previous trial was left in the wire cup-like cage (familiar mouse), while the object was replaced with a conspecific mouse (novel mouse). The test mouse was held in the middle chamber while the conspecific mouse and object were placed in the cup wire-like cages. The location of the conspecific mice and object were systemically altered in-between test mice. The three-chamber test apparatus and wire cup-like cages were cleaned with 70% ethanol after each test mouse and left to dry for a few minutes. To reduce potential anxiogenic factors, all mice were habituated to the testing room 40 min before the test, the floor of the testing arena was covered with sawdust and testing was performed under dim light (60 lux). All experiments were videotaped using a ceiling-mounted camera and were scored blinded for the time interacted with the wire cup-like cages. The discrimination index was calculated as follows: Time spent interacting with object or mouse/total time spent interacting × 100%.

### Elevated plus maze

The elevated plus maze test was used to assess anxiety-like behaviour and was conducted as previously described [14]. The elevated plus maze apparatus was elevated 1 m above the ground and consisted of a grey cross-shaped maze with two open arms and two closed arms (50 × 5 cm with 15 cm walls in the closed arms and 1 cm walls in the open arms). Mice were allowed to explore the maze for 5 min. Mice were habituated to the room 30 min prior to the test. Experiments were conducted in red light (5 lux). The elevated plus maze apparatus was cleaned with 70% ethanol in-between animals. Experiments were videotaped using a ceiling-mounted camera and videos were scored blinded for time spent in the open arms, which was defined as all paws in the open arm.

### Open-field test

Mice were assessed for locomotor activity and response to a novel environment in the open-field test, which was conducted as previously described [14]. Animals were placed in an open arena (40 × 32 × 24 cm, L × W × H) and were allowed to explore the arena for 10 min. Animals were habituated to the room 30 min prior to the test. Testing was performed under dim light (60 lux). The open field test box was cleaned with 70% ethanol in-between animals. Experiments were videotaped using a ceiling-mounted camera and were analysed for time spent in the virtual centre zone (defined as 50% away from the edges) and total distance travelled using Ethovision version 13 software (Noldus).

### Tail-suspension test

The tail-suspension test was used to assess depressive-like behaviour and was conducted as previously described [14]. Mice were hung by their tail using adhesive tape (2 cm from the tip of the tail) to a 30-cm-elevated grid bar for 6 min. Experiments were videotaped using a numeric tripod-fixed camera and videos were scored blinded for the time mice spent immobile.

### Saccharin preference test

Mice were assessed for reward-seeking behaviour using the saccharin preference test as previously conducted [54]. Mice were first habituated to single housing and having two drinking water bottles for 3 days. Drinking water intake and food intake was measured during the habituation phase of the test. Hereafter, one drinking water bottle was replaced by one containing a saccharin solution (0.1% w/v) for 24 h. Drinking water bottles were weighed every 12 h during the testing phase to calculate saccharin preference. The side on which the regular drinking water bottle and the one containing saccharine solution were randomised and counterbalanced between groups. During the habituation phase, drinking water bottles were alternated every 24 h, whereas bottles were alternated every 12 h during the testing phase. Saccharin preference was calculated using the following formula: Total sucrose intake/total fluid intake × 100%.

### Female urine sniffing test

Mice were assessed for hedonic and reward-seeking behaviour in the female urine sniffing test, which was performed as previously described [28]. Prior to this experiment, vaginal smears from age-matched female C57BL/6 mice (*n* = 20; Envigo, UK) were taken and assessed for their estrous cycle. Urine from female mice in the esterus stage was collected and pooled. Male mice were habituated 45 min before the start of the test to the test room, with a cotton bulb attached to the lid of their housing cage. The test mice were subsequently introduced to a new cotton bulb containing 60 μl sterile water. After a 45-min intertrial-interval, mice were introduced to a new cotton bulb containing 60 μl urine from a female mouse in esterus for 3 min. The experiment was conducted in red light (5 lux). All tests were videotaped using a ceiling-mounted camera and interaction time with the cotton bulbs was scored blinded.

### Stress-induced hyperthermia test

The stress-induced hyperthermia test was used to assess stress-responsiveness, which was conducted as previously described [14]. Body temperature was determined at baseline (T1) and 15 min later (T2) by gently inserting a Vaseline-covered thermometer 2.0 cm into the rectum. The temperature was noted to the nearest 0.1 °C after it stabilised (~ 10 s). Mice were restrained by scruffing during this procedure which was the stressor. Animals were habituated to the testing room 1 h prior to the test. The difference between T1 and T2 reflected the stress-induced hyperthermia.

### Appetitive Y-maze

The appetitive Y-maze was used to assess long-term spatial learning and was performed as previously described [27]. The test consisted of two phases; the initial learning phase, where the first association between the location of the food reward and spatial reference cues were formed, and the reversal learning phase, where the location of the food reward was altered in reference to the spatial reference cues, in which the relearning of a context was measured.

The Y-maze apparatus was elevated 80 cm above the ground and consisted of three arms (50 × 9.5 cm, L × W, with a 0.5-cm-high rim) arranged at an angle of 120° of each other (Figure S10A). The apparatus could be rotated during testing. A small plastic food well (a cap of a 15-mL tube) was placed at the distal end of each arm. Testing was performed under dim light (30 lux).

Prior to testing, mice were food-restricted (3–4 g food per day) and kept between 90 and 95% of their free-feeding body weight (Figure S10B). Two days later, animals were habituated in their home cage to the small plastic well containing 1 mL food reward (sweetened condensed milk diluted in water 1:1) per mouse before the onset of the active phase. Mice were subsequently habituated on the Y-maze apparatus in home cage groups until mice were freely running around and readily collecting the food reward (each arm contained 1 mL food reward), which took 2 days. Finally, mice were individually placed on the Y-maze until they were running and collected the food reward (each arm contained 0.1 mL food reward), which took 4 days.

During the first phase (Initial learning), mice were assigned a goal arm according to the position in the room, which was counter-balance between groups. The maze was rotated 120° every trial to prevent potential associations of the correct goal arm with the texture or smell of the arm. The starting position for each trial was determined by a pseudorandomised computer sequence, which was different for each mouse but was the same across treatment groups. This sequence did not contain more than three consecutive starts in the same position to avoid temporary position preferences. Animals were tested in groups of eight, with four animals per two experimental group (i.e. two home cages). Each mouse received ten trials per day with an intertrial interval of approximately 10 min. The time of testing was counterbalanced between groups and rotated each day to reduce the effect of testing during a specific time of the day. Mice received eight consecutive days of initial learning, resulting in a total of 80 trials. During the second phase (reversal learning), the goal arm was changed to a different arm, and the placement of the mice was changed accordingly. This phase lasted 5 days, resulting in a total of 50 trials.

For each trial, the food well on the goal arm was filled with 0.1 mL food reward (sweetened condensed milk diluted in water 1:1). The mouse was placed at the end of the start arm and was allowed to run freely on the maze. The entries into each arm were counted, as well as when the mouse went into the goal arm immediately, of which the latter was counted as a successful trial. The mouse was placed back into the home cage after it consumed the food reward. In the rare occasion that the mouse did not walk into the goal arm and collect the food reward within 90 s, then the mouse was gently guided towards the goal arm and given a chance to collect the food reward, after which it was also returned to the home cage. A trial where the mouse did not walk into any arm was excluded from the analysis, as this indicates that the mouse was anxious. An entry was counted when the tail of the animal passed the entry of the arm. Between mice, the food wells were not cleaned so that a slight odour of milk reward remained at all times, ensuring mice found the goal arm based on spatial cues, rather the olfactory cues.

### Fear conditioning

Fear conditioning was used to assess amygdala-dependent learning memory and was conducted as previously described [35]. The test consisted of 3 days/phases: (1) training, (2) assessment of cued memory, (3) assessment of contextual memory, each of which was carried on successive days with a 24-h interval. In phase 1 (training), animals were recorded for 3 min (baseline), followed by 6 tone-conditioned stimuli (70 dB, 20 s), followed by a foot shock (0.6 mA, 2 s), with a 1-min interval. In phase 2 (assessment of cued memory), mice were placed in a novel context (i.e. black-checkered walls with a solid Plexiglas opaque floor, under which paper was placed containing a 400 μl vanilla solution (79.5% water/19.5% ethanol/1% vanilla-extract solution), and after an initial acclimation period of 2 min, mice received 40 presentations of the tone-conditioned stimuli, each lasting 30 s with a 5-s interval. In phase 3 (assessment of contextual memory), mice were placed in the context of day 1 and recorded for 5 min, without the presentation of any tone-conditioned stimuli. The fear conditioning apparatus was cleaned with 70% ethanol in-between animals.

### Forced swim test

The forced swim test was used to assess depressive-like behaviour and was conducted as previously described [19]. Mice were individually placed in a transparent glass cylinder (24 × 21 cm diameter) containing 15-cm-depth water (23–25 °C) for 6 min. Mice were gently dried after the test, and water was renewed after each animal. Experiments were videotaped using a ceiling-mounted camera and videos were scored blinded for immobility time in the last 4 min of the test.

### Repeated plasma sampling for corticosterone quantification

Plasma from each animal was sampled by tail-tip 5 min before the forced swim test, and repeatedly after the test in 30 min intervals up to 120 min, as previously discussed [14]. For the tail-tip, the end of the tail was gently held with two fingers without restraining the mouse. Using a single edge razor blade, a 2–4 mm long diagonal incision was made at the end of the tail. Approximately, 40 μl of whole blood was taken per time point using an EDTA-containing capillary (Fisher Scientific, 749311), deposited in an Eppendorf and centrifuged for 10 min at 3500×*g* at 4 °C. Plasma was collected and stored at −80 °C for later corticosterone quantification.

### Intestinal motility assay

Gastrointestinal motility was assessed as previously described [31]. Briefly, mice were singly housed at 8.00 am. with *ad libitum* access to food and drinking water. Three hours later, 0.2 mL of non-absorbable 6% carmine red in 0.5% methylcellulose dissolved in sterile phosphate-buffered saline was administered by oral gavage, after which drinking water was removed. The latency for the excretion of the first red-coloured faecal pellet was subsequently timed as a measure of gastrointestinal motility.

### Assessment of faecal water content and weight

Mice were singly housed for 1 h during which faecal pellets were collected (± 9 per animal). Pellets were subsequently weighed, dried at 50 °C for 24 h and weighed again. The average weight per pellet and percentage of faecal water content was calculated.

### Tissue collection

Collection of faecal samples for metabolomics was done one week prior to euthanasia. This was done by single housing mice until 2 pellets were dropped between 10.00 and 12.00 am. The order of faecal pellet collection was counterbalanced between groups to minimise the effect of circadian rhythm. Pellets were snap-frozen on dry ice within 3 min after excretion and subsequently stored at −80 *°C.*

Animals were sacrificed by decapitation in a random fashion regarding test groups between 9.00 am and 2.00 pm. Trunk blood was collected in 3 mL EDTA-containing tubes (Greiner bio-one, 454086) and 100 μl was put in a separate Eppendorf for flow cytometry. Both tubes were centrifuged for 10 min at 3500×*g* at 4 °C, after which plasma was collected and stored at –80 °C for cytokine quantification. The remaining cell pellet of the Eppendorf containing 100 μl blood was stored on ice and subsequently used for flow cytometry. Mesenteric lymph nodes (MLNs) were extracted, fat tissue was removed and stored in RPMI-1640 medium with l-glutamine and sodium bicarbonate (R8758, Sigma), supplemented with 10% FBS (F7524l, Sigma) and 1% Pen/strep (P4333, Sigma) on ice for subsequent flow cytometry. The faecal pellets, caecum, and contents of the distal part of the ileum (2 cm) were collected, snap-frozen on dry ice and stored at –80 °C for shotgun sequencing.

### Flow cytometry

Blood and MLNs collected when animals were sacrificed were processed on the same day for flow cytometry, as previously described [8, 32]. Blood was resuspended in 10 mL home-made red blood cell lysis buffer (15.5 mM NH_4_Cl, 1.2 mM NaHCO_3_, 0.01 mM tetrasodium EDTA diluted in deionised water) for 3 min. Blood samples were subsequently centrifuged (1500×*g*, 5 min), split into 2 aliquots and resuspended in 45 μl staining buffer (autoMACS Rinsing Solution (Miltenyi, 130-091-222) supplemented with MACS BSA stock solution (Miltenyi, 130-091-376)) for the staining procedure. MLNs were poured over a 70 μm strainer and disassembled using the plunger of a 1-mL syringe. The strainer was subsequently washed with 10 mL media (RPMI-1640 medium with l-glutamine and sodium bicarbonate, supplemented with 10% FBS and 1% Pen/strep), centrifuged and 2 × 10^6^ cells were resuspended in 90 μl staining buffer and split into two aliquots for the staining procedure. For the staining procedure, 5 μl of FcR blocking reagent (Miltenyi, 130-092-575) was added to each sample. Samples were subsequently incubated with a mix of antibodies (Blood aliquot 1; 5 μl CD11b-VioBright FITC (Miltenyi, 130-109-290), 5 μl LY6C-PE (Miltenyi, 130-102-391), 0.3 μl CX3CR1-PerCP-Cyanine5.5 (Biolegend, 149010) and 5 μl CCR2-APC (Miltenyi, 130-108-723); Blood aliquot 2 and MLNs 1; 1 μl CD4-FITC (ThermoFisher, 11-0042-82) and 1 μl CD25-PerCP-Cyanine5.5 (ThermoFisher, 45-0251-80); MLNs 2; 5 μl CD103-FITC (Miltenyi, 130-102-479), 2 μl CD11c-PE (Miltenyi, 130-110-838), 0.3 μl CX3CR1-PerCP-Cyanine5.5 (Biolegend, 149010) and 5 μl MHC-II-APC (Miltenyi, 130-102-139)) and incubated for 30 min on ice. Blood aliquot 1 was subsequently fixed in 4% PFA for 30 min on ice, whilst Blood aliquot 2 and MLNs underwent intracellular staining using the eBioscience™ Foxp3/Transcription Factor Staining Buffer Set (ThermoFisher, 00-5523-00), according to the manufacturers’ instructions, using antibodies for intracellular staining (2 μl FoxP3-APC (ThermoFisher, 17-5773-82) and 5 μl Helios-PE (ThermoFisher, 12-9883-42)). Fixed samples were resuspended in staining buffer and analysed the subsequent day on the BD FACSCalibur flow cytometry machine. Data were analysed using FlowJo (version 10), see Figure S11 for the gating information. Cell populations were selected as following: Treg cells: CD4+, CD25+, FoxP3+; Neutrophils: CD11b+, LY6C^mid^. SSC^high^; Monocytes: CD11b+, LY6C^high^; CD103+ Dendritic cells; MHC-II+, CD11c+, CD103+. The investigated cell populations were normalised to PBMC levels.

### Plasma corticosterone and cytokine assessment

Corticosterone quantification of plasma samples (20 μl) obtained in the forced swim test was performed using a corticosterone ELISA (Enzo Life Sciences, ADI-901-097) according to the manufacturer’s guidelines. A multi-mode plate reader (Synergy HT, BioTek Instruments) was used to measure light absorbance. Cytokine levels from plasma samples collected during euthanasia were quantified using the V-PLEX Proinflammatory Panel 1 Mouse Kit (MSD, K15048D). Cytokine quantification was done according to the manufacturer’s guidelines with one modification, where 20 μl plasma sample was added onto the plate and incubated overnight (15 h) at 4 °C, after which the rest of the protocol was carried out as suggested by the guidelines. Values under the fit curve range and detection range were excluded.

### High-performance liquid chromatography

5-hydroxytryptamine (5-HT) and 5-hydroxyindoleacetic acid (5-HIAA) concentrations were determined using HPLC based on methodology previously described [16]. Briefly, mobile phase consisted of HPLC-grade 0.1 M citric acid, 0.1 M sodium dihydrogen phosphate monohydrate, 0.01 mM EDTA disodium salt (Alkem/Reagecon), 5.6 mM octane-1-sulphonic acid (Sigma Aldrich), and 9% (v/v) methanol (Alkem/Reagecon). The pH of the mobile phase was adjusted to 2.8 using 4 N sodium hydroxide (Alkem/Reagecon). Homogenisation buffer consisted of mobile phase with the addition of 20 ng/20 μl of the internal standard, N-methyl 5-HT (Sigma Aldrich). Briefly, tissue samples were sonicated (Sonopuls HD 2070) for 4 s in 500 μl cold homogenisation buffer during which they were kept chilled. Tissue homogenates were then centrifuged at 14,000×*g* for 20 min at 4 °C. The supernatant was collected and the pellet was discarded. The supernatant was then briefly vortexed and 30 μl of supernatant was spiked into 270 μl of mobile phase. Twenty microliters of the 1:10 dilution was injected into the HPLC system (Shimadzu, Japan) which was comprised of a SCL 10-Avp system controller, LC-10AS pump, SIL-10A autoinjector, CTO-10A oven, LECD 6A electrochemical detector, and Class VP-5 software. The chromatographic conditions were flow rate of 0.9 mL/min using a Kinetex 2.6 u C18 100 A × 4.6 mm column (Phenomenex), oven temperature of 30 °C, and detector settings of + 0.8 V. 5-HT and 5-HIAA external standards (Sigma Aldrich, H7752 and H8876, respectively) were injected at regular intervals during sample analyses. Monoamines in unknown samples were determined by their retention times compared to external standards. Peak heights of the analyte: internal standard ratio were measured and compared with external standards, results were expressed as microgram of neurotransmitter per gram of tissue.

### DNA extractions and sequencing

For analysis of the kefir microbiome, DNA was extracted from the fermented milk using the PowerSoil DNA Isolation Kit, as described previously [88]. For analysis of the murine gut microbiome, DNA was extracted from the total ileal contents, caecal contents and faecal pellets using the QIAamp PowerFaecal DNA Kit. Whole-metagenome shotgun libraries were prepared using the Nextera XT kit in accordance with the Nextera XT DNA Library Preparation Guide from Illumina, with the exception that tagmentation time was increased to 7 min. Kefir libraries were sequenced on the Illumina MiSeq sequencing platform with a 2 × 300 cycles v3 kit. Gut libraries were sequenced on the Illumina NextSeq 500 with a NextSeq 500/550 High Output Reagent Kit v2 (300 cycles). All sequencing was performed at the Teagasc sequencing facility in accordance with standard Illumina protocols.

### Faecal metabolomics

The faecal metabolome was analysed by chromatography–mass spectrometry (GC-MS) by MS-Omics, Copenhagen. Samples were derivatized using methyl chloroformate. For SCFA quantification, samples were acidified with hydrochloric acid.

### Bioinformatics

Murine reads were removed from the raw sequencing files using the NCBI Best Match Tagger (BMTagger) (ftp://ftp.ncbi.nlm.nih.gov/pub/agarwala/bmtagger/), and fastq files were converted to unaligned bam files using SAMtools [43]. Duplicate reads were subsequently removed using Picard Tools (https://github.com/broadinstitute/picard). Next, low-quality reads were removed using the trimBWAstyle.usingBam.pl script from the Bioinformatics Core at UC Davis Genome Center (https://github.com/genome/genome/blob/master/lib/perl/Genome/Site/TGI/Hmp/HmpSraProcess/trimBWAstyle.usingBam.pl). Specifically, MiSeq reads were filtered to 200 bp, while NextSeq were filtered to 105 bp. All reads with a quality score less than Q30 were discarded. The resulting fastq files were then converted to fasta files using the fq2fa option from IDBA-UD [59].

Compositional analysis was performed using MetaPhlAn2 [83]. Strain-level metagenomic analysis was performed using StrainPhlAn [84] and PanPhlAn [66] StrainPhlAn outputs were visualised using GraPhlAn [4]. Custom PanPhlAn databases were constructed from complete genome assemblies which were annotated using Prokka [67]. See Table S1 for the list of reference genomes used in this study. Functional analysis was performed with HUMAnN2 [2], using the bypass-translated-search option, and PanPhlAn. HUMAnN2 gene families were mapped to level-4 enzyme commission (EC) categories using HUMAnN2 utility mapping files. Sequence data have been deposited in the European Nucleotide Archive (ENA). Correlations between gut microbial species and significantly altered behavioural and immunological parameters were investigated using HAllA (https://bitbucket.org/biobakery/halla/wiki/Home).

Analysis of GBMs was performed as previously described [85]. Briefly, the UniRef gene families that were detected by HUMAnN2 were mapped to KEGG Orthogroups (KOs) using the humann2_regroup_table function, and the abundances of KOs were normalised using the humann2_renorm_table function. Next, these KOs were further mapped to GBMs using Omixer-RPM.

Metagenome co-assembly was performed using MEGAHIT [42]. MetaBAT 2 [37] was used to recover genomes from the metagenome. CheckM [57] was used to assess the quality of the MAGs. The *Lactobacillus reuteri* genome was identified using PhyloPhlAn [68]. Prokka was used to annotate the genome and CarveMe [49] was used to construct a metabolic model. COBRApy [26] was used to perform flux variability analysis (FVA), with an objective of 95% biomass, of this model.

Raw microbiota reads have been deposited to the European Nucleotide Archive under the project accession number PRJEB35751.

### Statistical analysis

All behavioural and physiological data were assessed for normality using the Shapiro-Wilk test and Levene’s test for equality of variances. The effect of kefir was determined by a one-way ANOVA, followed by Dunnett’s post hoc test whenever data were normally distributed. If data were non-parametrically distributed, then a Kruskal-Wallis test followed by a Mann-Whitney *U* test was used. Undisturbed control and milk control datasets were assessed for statistical significance using an unpaired Student’s *t* test or a Mann-Whitney *U* test to investigate the impact of milk gavage. Bodyweight, fear conditioning and appetitive Y-maze data were assessed using repeated-measures ANOVA, followed by a Dunnett’s post hoc test. The presence of social preference and recognition in the 3-chamber sociability test was assessed using a paired Student’s *t* test. Parametric data are depicted as bar graphs with points as individual data points and expressed as mean ± SEM. Non-parametric data is depicted as a box with whiskers plot. Statistical analysis was performed using SPSS software version 24 (IBM Corp). A *p* value < 0.05 was deemed significant

Statistical analysis for bioinformatics data was performed using the R package vegan for alpha diversity analysis and principal component analysis [56]. The Wilcoxon rank-sum test was used to measure statistical differences in alpha diversity between groups. The adonis function from vegan was used for PERMANOVA. The linear discriminant analysis (LDA) effect size (LEfSe) method [69] was used to investigate if any taxa or HUMAnN2 pathways were differentially abundant between groups. Data were visualised using hclust2 (https://bitbucket.org/nsegata/hclust2), GraPhlAn, and the R package ggplot2 [89].

## Supplementary information

**Additional file 1.**

## Data Availability

Raw microbiota reads have been deposited to the European Nucleotide Archive under the project accession number PRJEB35751.

## Abbreviations

Treg: T regulatory cell
MLN: Mesenteric lymph node
pTreg: Peripheral-derived T regulatory cells
5-HT: Serotonin
5HIAA: 5-hydroxyindoleacetic acid
SCFA: Short-chain fatty acid
GC-MS: Chromatography–mass spectrometry
EC: Enzyme commission
GBM: Gut-brain modules
KO: KEGG Orthogroup
GABA: Gamma aminobutyric acid
