# Supplementary material for: Distinct actions of the fermented beverage kefir on host behaviour, immunity and microbiome gut-brain modules in the mouse
Source: Microbiome. 2020 May 18;8:67. doi: 10.1186/s40168-020-00846-5 (PMC7236220; doi:10.1186/s40168-020-00846-5)
Supplement: Supplementary file 2 — Additional file 1. [file 40168_2020_846_MOESM1_ESM.docx]

# Supplementary information

# Distinct Actions of the Fermented Beverage Kefir on Host Behaviour, Immunity and Microbiome Gut-Brain Modules in the Mouse

Marcel van de Wouw^1,2*^, Aaron M. Walsh^1,3,5*^, Fiona Crispie^1,3^, Lucas van Leuven^1^, Joshua M. Lyte^1^, Marcus Boehme^1^, Gerard Clarke^1,4^, Timothy G. Dinan^1,4^, Paul D. Cotter^1,3$^, John F. Cryan^1,2,4$^

^*^Shared first co-author

^$^Shared last co-author

^1^APC Microbiome Ireland, University College Cork, Cork, Ireland

^2^Department of Anatomy and Neuroscience, University College Cork, Cork, Ireland

^3^Teagasc Food Research Centre, Moorepark, Fermoy, Cork, Ireland.

^4^Department of Psychiatry and Neurobehavioral Science, University College Cork, Cork, Ireland

^5^Microbiology Department, University College Cork, Cork, Ireland

Corresponding authors:

J.F. Cryan: APC Microbiome Ireland; Department of Anatomy and Neuroscience, University College Cork, Cork, Ireland. Email: [j.cryan@ucc.ie](mailto:j.cryan@ucc.ie).

P.D. Cotter: APC Microbiome Ireland; Teagasc Food Research Centre, Moorepark, Fermoy, Co. Cork, Ireland. Email: [paul.cotter@teagasc.ie](mailto:paul.cotter@teagasc.ie)


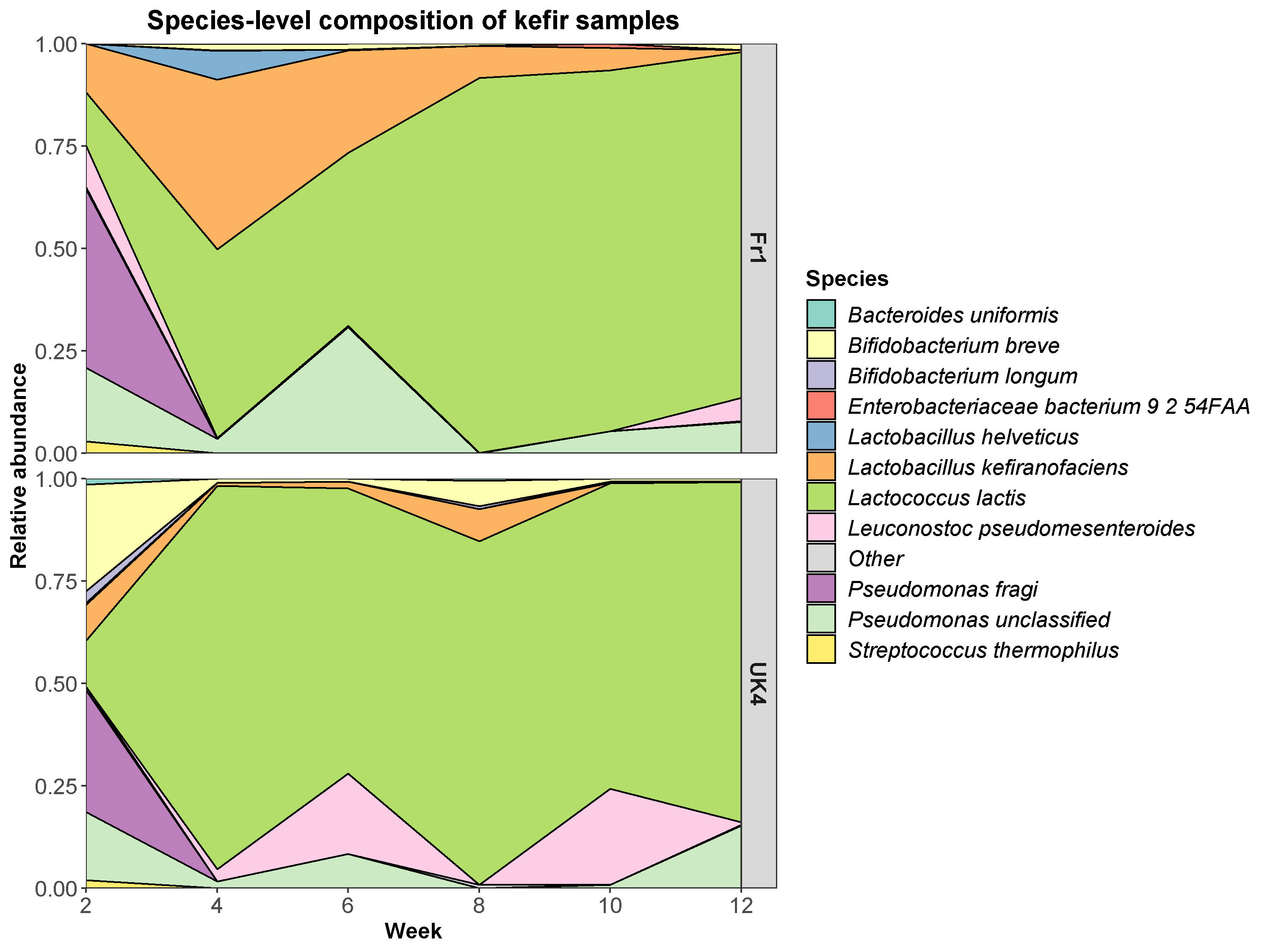


***Figure S1.*** *Stacked area chart showing the microbial composition of kefirs over the course of the experiment.*





***Figure S2. Kefir was well-tolerated in mice.***  *Body weight as measured throughout the study (A). The gap in-between day 64 and 92 represents the appetitive Y-maze, in which animals were food restricted. Food intake and drinking water intake were measured during the habituation phase of the saccharin preference test (B, C). Body composition (i.e. lean, fat and fluid mass) were quantified at the end of the study (D-F). Basal body temperature was taken during the stress-induced hyperthermia test (G). Locomotor activity was assessed in the open field test. All data are expressed as mean ± SEM (n = 11-12). Dots on each graph represent individual animals.*





***Figure S3. Kefir did not influence gastrointestinal motility.*** *Gastrointestinal motility was assessed by carmine red administration (A). Faecal pellet weight and water content were quantified during the “faecal water content assessment” (B, C). Caecum weight and colon length were measured at the end of the study (D, E). All data are expressed as mean ± SEM (n = 11-12). Dots on each graph represent individual animals.*


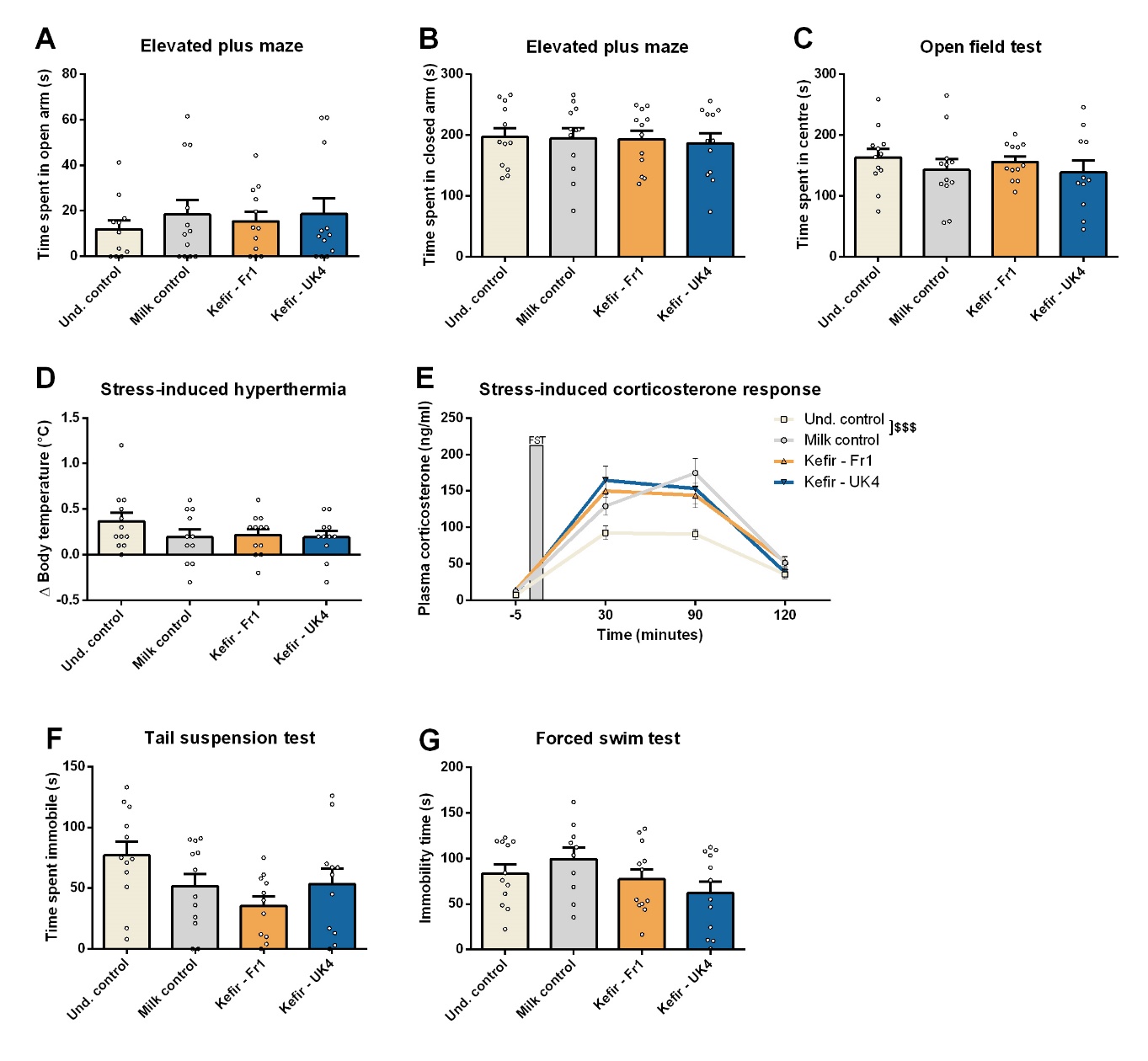


***Figure S4. Selective anxiety-like and depressive-like behavioural measurement showed no differences.*** *Repetitive/anxiety-like behaviour was assessed using the elevated plus maze and open field test (A, B, C). Stress-responsiveness was determined using the stress-induced hyperthermia test and by measuring corticosterone levels in response to an acute stressor (D,E). Depressive-like behaviour was investigated using the tail suspension test and forced swim test (F, G). Interestingly, the Milk control group did show an increase in stress-induced corticosterone response (F(1,20) = 15.936, p < 0.001), which was analysed using a repeated measures ANOVA. All data are expressed as mean ± SEM (n = 11-12). Dots on each graph represent individual animals.*





***Figure S5. Kefir did not influence social preference or recognition.*** *Social preference and recognition were assessed with the 3-chamber social interaction test (A, B). The presence of social preference and recognition were assessed using a paired Student’s t-test. All data are expressed as mean ± SEM (n = 12).*


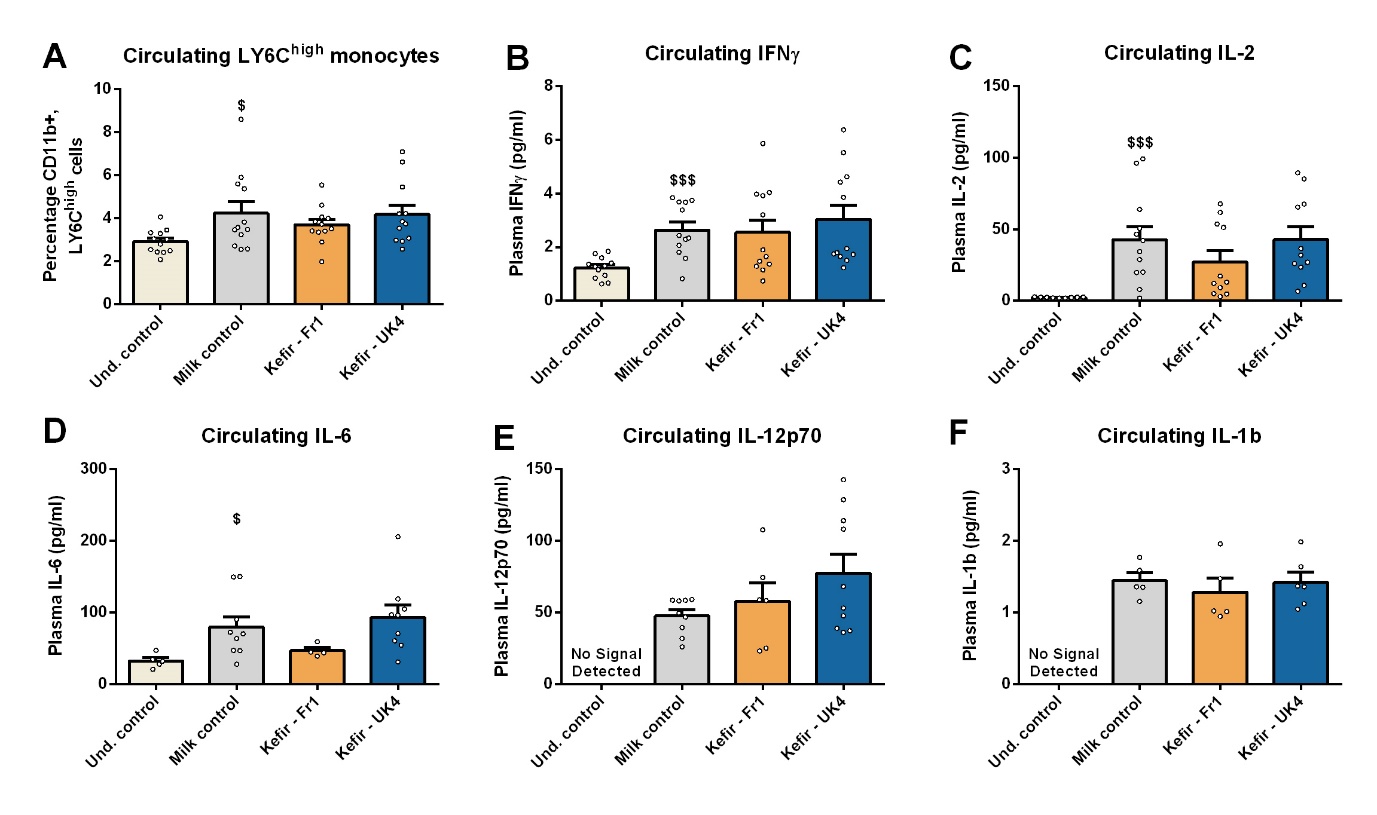


***Figure S6. Mice receiving milk gavage show a low-grade systemic inflammation.*** *LY6C_high_ monocytes were investigated at the end of the study using flow cytometry (A), see* ***figure S2*** *for gating. Plasma cytokines (i.e. IL-2, IL-6, IL-12p70, IL-1β) were also quantified at the end of the study (B-F). Differences between Und. Control and Milk control were analysed using an unpaired Student’s t-test. The Milk control groups showed elevated levels of LY6C^high^ monocytes (t(22) = −2.437, p = 0.023), IFNγ (t(22) = −4.514, p < 0.001), IL-2 (t(19) = −3.838, p < 0.001), and IL-6 (t(12) = −2.354, p = 0.036). Similarly, no IL-12p70 and IL-1β were detected in the Und control group but were detected in several samples of the other groups. Values under the fit curve and detection limit were excluded from the cytokine analysis. All data are expressed as mean ± SEM. Dots on each graph represent individual animals*


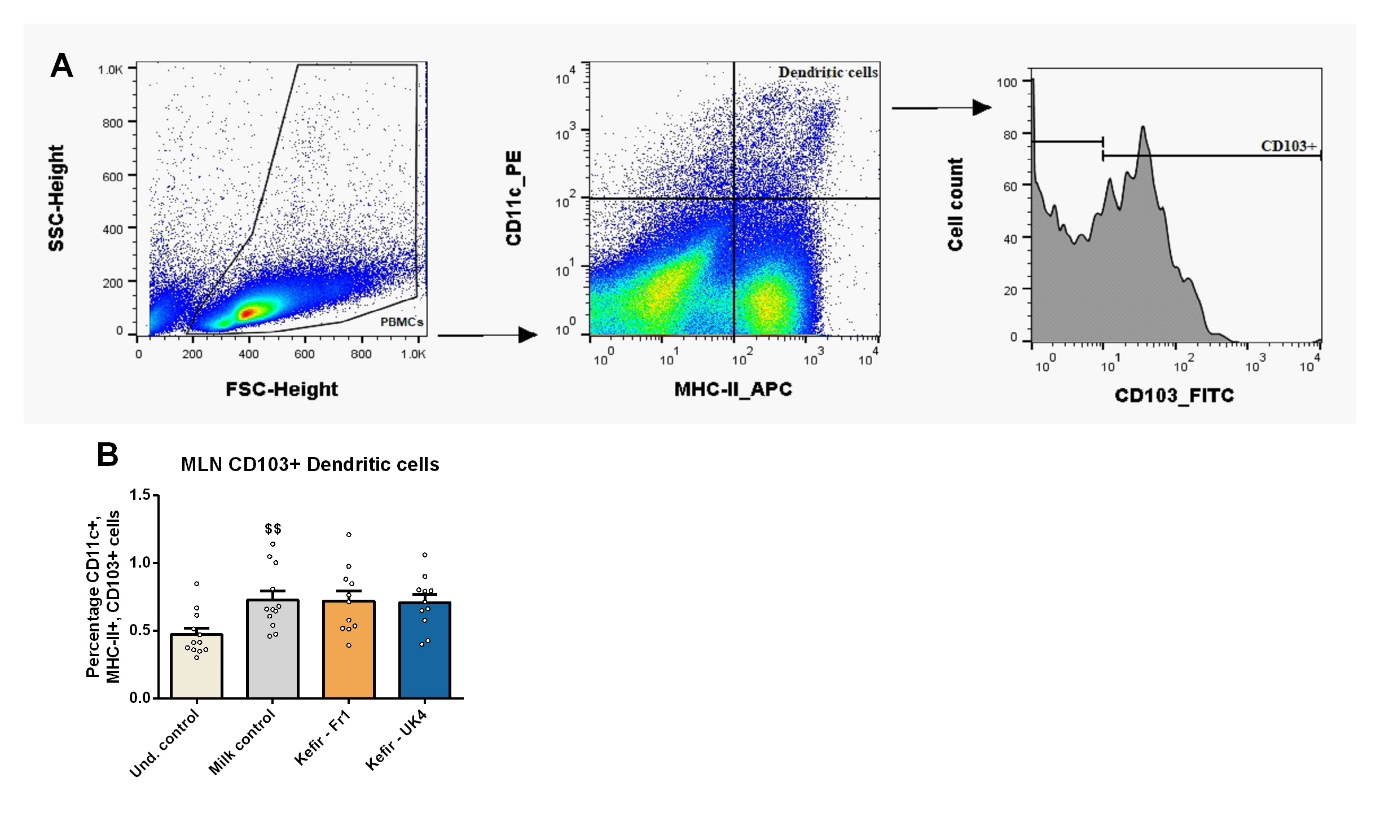


***Figure S7. Kefir administration did not affect the prevalence of MLN CD103+ Dendritic cells.*** *Mesenteric lymph node (MLN) CD103+ dendritic cells (CD11c+, MHC-II+, CD103+) were quantified using flow cytometry (A, B). Interestingly, the Milk control group did show increased levels of MLN CD103+ dendritic cells (t(22) = −3.165, p = 0.004), as analyzed by an unpaired Student’s t-test (B). All data are expressed as mean ± SEM (n = 12). Dots on each graph represent individual animals.*


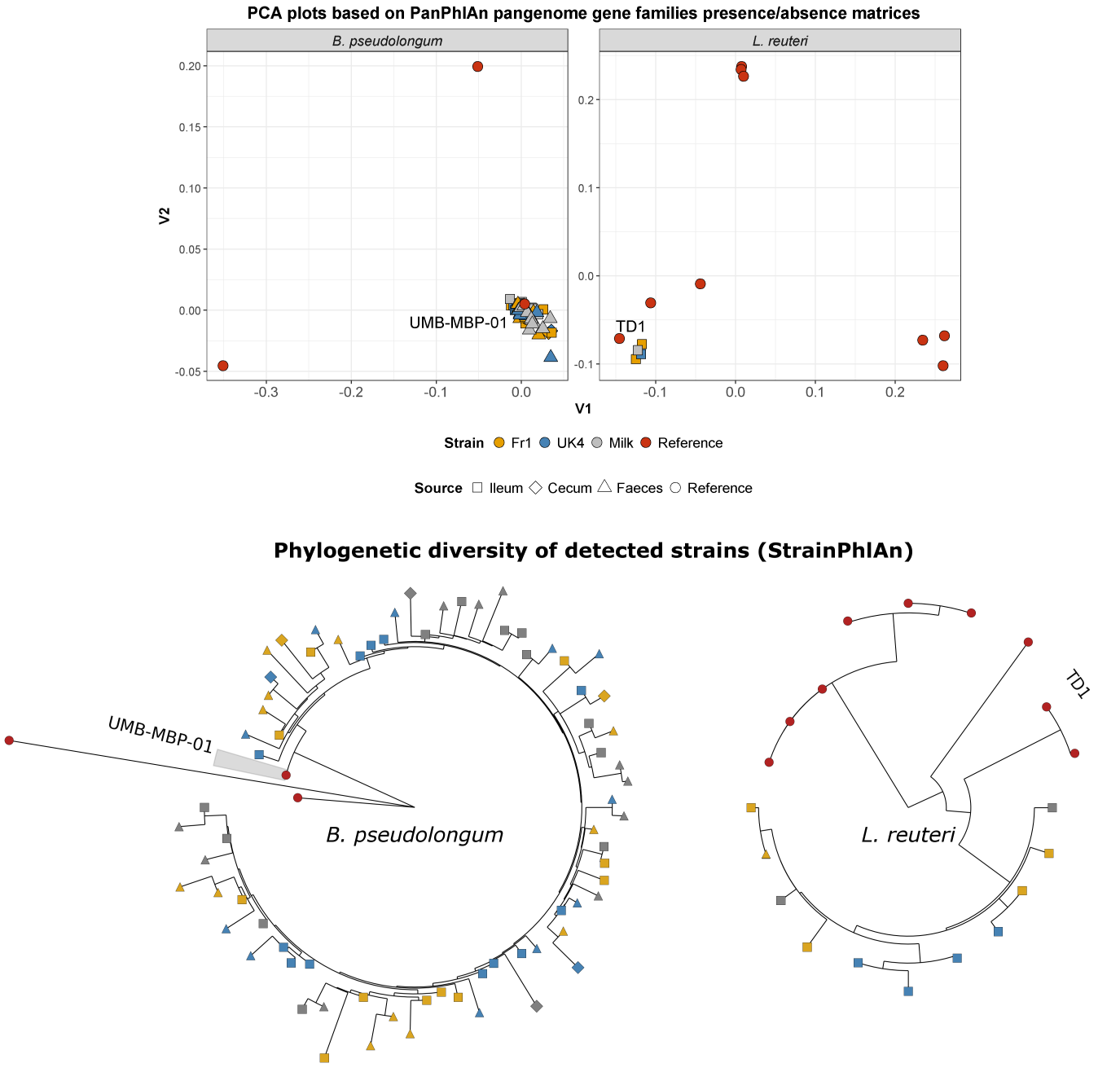


***Figure S8. Strain-level analysis of Bifidobacterium pseudolongum and Lactobacillus reuteri detected in the mouse gut.*** *(A) PCA plot based on gene families presence/absence matrices from PanPhlAn. The reference strains which shared the most gene families with that detected in the murine gastrointestinal tract are labelled. (B) Phylogenetic trees generated from StrainPhlAn outputs. Note that colours represent the group to which strains belong and shapes represent the source of the strains.*



 ***Figure S9: Faecal short-chain fatty acid levels were unaffected by kefir.*** *The presence of enzymes involved in SCFA metabolism were detected in the kefir (e.g. Acetoacetyl-CoA reductase (EC: 1.1.1.36), Acetyl-CoA C-acetyltransferase (EC: 2.3.1.9), and Aconitate hydratase (EC: 4.2.1.3)) (data not shown). We subsequently analyzed the three principle SCFAs (acetate, propionate and butyrate) by chromatography–mass spectrometry, where no differences were observed. In addition, no changes were found in the predicted levels of SCFA-metabolizing enzymes in the gastrointestinal microbiota of mice receiving kefir. All data are expressed as mean ± SEM (n = 6). Dots on each graph represent individual animals.*



 ***Figure S10. Room layout with cues for the appetitive Y-maze and food restriction.*** *The room layout with the various cues used in the appetitive Y-maze is depicted (A). Mice were kept on food restriction of 90-95% of the free-feeding body weight. All data are expressed as mean ± SEM (n = 12).*


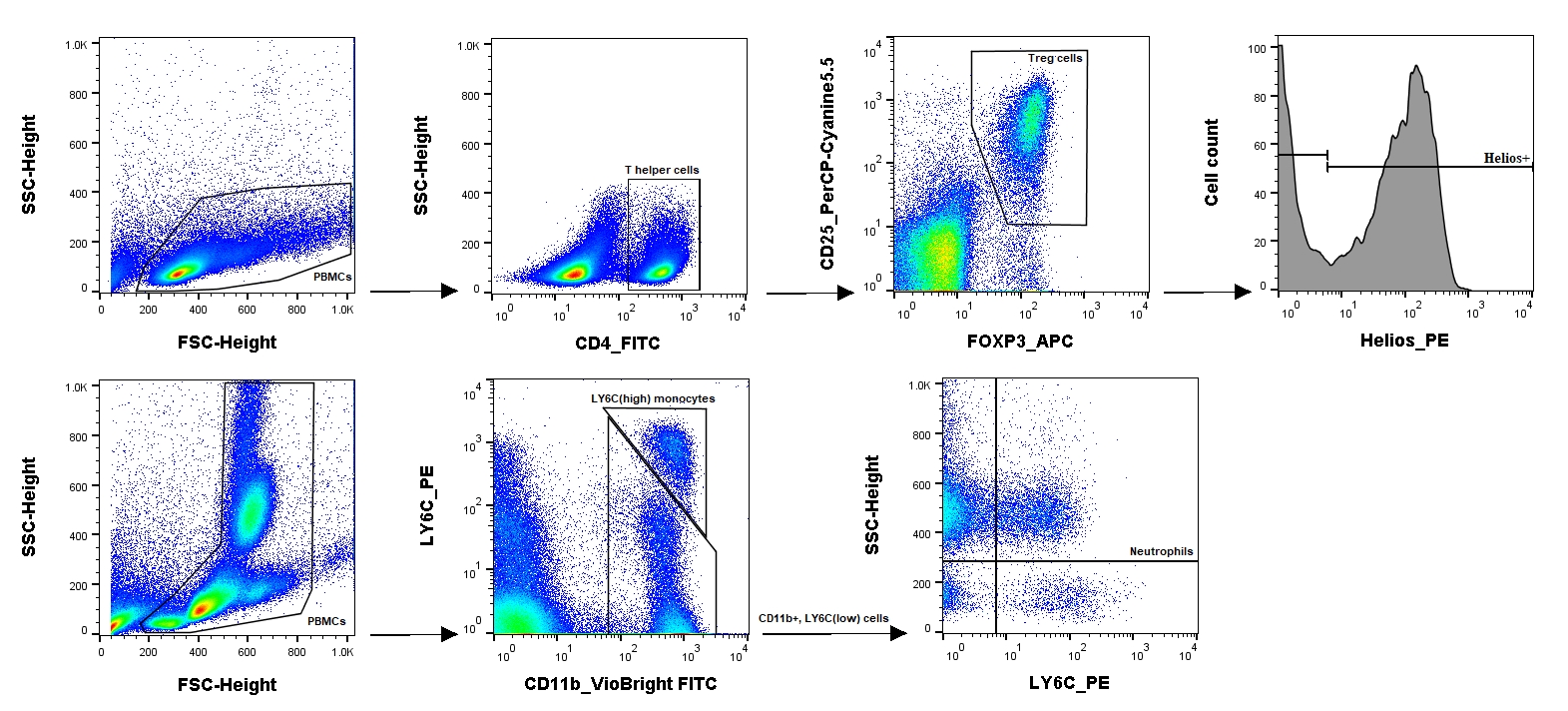


***Figure S11: Flow cytometry gating of Treg cells, inflammatory monocytes and neutrophils.*** *Cells (PBMCs) were selected based on FSC-height and SSC-height. The top four figures show the gating strategy for T regulatory (Treg: CD4+, CD25+, FOXP3+), whereas the bottom 3 graphs show the gating for monocytes (CD11b+, LY6C^high^) (F) and neutrophils (CD11b+, LY6C^mid^, SSC^high^). T helper cells (CD4+) were subsequently selected based on CD4 receptor expressions and Treg cells were selected on CD25 receptor expression and FOXP3 transcription factor expression Finally, cells were analysed for the expression of the transcription factor Helios, as measure of their origin (i.e. periphery (pTreg). Monocytes were selected using LY6C and CD11b expression. Neutrophils were gated by first selecting CD11b+, LY6C^-/mid^ cells, after which SSC^high^, LY6C^mid^ cells were selected.*

**Table S1: Reference genomes which were included in the custom PanPhlAn pangenome databases used in this study.**

| **Species** | **Assembly** | **Strain** |
| --- | --- | --- |
| *Lactobacillus reuteri* | GCF_000010005 | JCM 1112 |
| *Lactobacillus reuteri* | GCF_000016825 | DSM 20016 |
| *Lactobacillus reuteri* | GCF_000159455 | SD2112 |
| *Lactobacillus reuteri* | GCF_000236455 | ATCC 53608 |
| *Lactobacillus reuteri* | GCF_000410995 | I5007 |
| *Lactobacillus reuteri* | GCF_000439275 | TD1 |
| *Lactobacillus reuteri* | GCF_001046835 | IRT |
| *Lactobacillus reuteri* | GCF_001618905 | ZLR003 |
| *Lactobacillus reuteri* | GCF_001688685 | I49 |
| *Bifidobacterium pseudolongum* | GCF_000800475 | PV8-2 |
| *Bifidobacterium pseudolongum* | GCF_002282915 | UMB-MBP-01 |
| *Bifidobacterium pseudolongum* | GCF_002706665 | DSM 20092 |

**Table S2: Enzyme Commission (EC) level 4 categories which were differentially abundant between kefir versus Milk-fed mice, as determined by LEfSe.**

**sTable 3. Faecal metabolomics.** The faecal metabolome was assessed by as chromatography–mass spectrometry. Differences between undisturbed control and milk control were assessed for statistical significance using an unpaired Student’s t-test. The effect of kefir compared to milk was determined by a one-way ANOVA, followed by Dunnett's post hoc test. Significant differences are depicted as: ^$^*p*< 0.05; Undisturbed control compared to milk control, **p*< 0.05; Milk control compared to kefir. All data are expressed as mean ± SEM (n = 6).

|  | Undisturbed Control | | Milk control | | Kefir - Fr1 | | Kefir - UK4 | |
| --- | --- | --- | --- | --- | --- | --- | --- | --- |
|  | Average (mM) | SEM | Average (mM) | SEM | Average (mM) | SEM | Average (mM) | SEM |
| Alanine | 0.65 | 0.15 | 0.51 | 0.18 | 0.58 | 0.24 | 0.75 | 0.37 |
| Glycine | 0.20 | 0.05 | 0.23 | 0.10 | 0.16 | 0.07 | 0.29 | 0.23 |
| Valine | 0.23 | 0.06 | 0.19 | 0.09 | 0.20 | 0.08 | 0.28 | 0.11 |
| Leucine | 0.22 | 0.05 | 0.19 | 0.10 | 0.19 | 0.07 | 0.26 | 0.09 |
| Isoleucine | 0.17 | 0.04 | 0.13 | 0.07 | 0.14 | 0.06 | 0.21 | 0.09 |
| Threonine | 0.14 | 0.02 | 0.11 | 0.05 | 0.14 | 0.04 | **0.18*** | 0.06 |
| Proline | 0.13 | 0.02 | 0.11 | 0.04 | 0.11 | 0.03 | **0.15*** | 0.04 |
| Asparagine | 0.02 | 0.00 | 0.03 | 0.03 | 0.02 | 0.01 | 0.03 | 0.01 |
| Glutamine | 0.44 | 0.21 | **0.17^$^** | 0.06 | 0.21 | 0.12 | 0.43 | 0.35 |
| Serine | 0.19 | 0.02 | 0.18 | 0.07 | 0.19 | 0.06 | 0.22 | 0.06 |
| Glutamic acid | 1.05 | 0.38 | 0.64 | 0.22 | 0.68 | 0.45 | 1.13 | 0.93 |
| Methionine | 0.07 | 0.03 | 0.05 | 0.03 | 0.06 | 0.03 | 0.09 | 0.06 |
| Phenylalanine | 0.10 | 0.03 | 0.08 | 0.04 | 0.09 | 0.03 | **0.13*** | 0.05 |
| Ornithine | 0.05 | 0.01 | **0.03^$^** | 0.01 | **0.05*** | 0.01 | 0.06 | 0.02 |
| Lysine | 0.36 | 0.10 | **0.22^$^** | 0.08 | 0.27 | 0.16 | **0.38*** | 0.21 |
| Histidine | 1.26 | 0.16 | **0.61^$^** | 0.23 | **0.98*** | 0.30 | 1.15 | 0.49 |
| Tyrosine | 0.10 | 0.03 | 0.08 | 0.03 | 0.09 | 0.04 | **0.12*** | 0.05 |
| Aspartic acid | 0.12 | 0.03 | 0.12 | 0.08 | 0.11 | 0.04 | 0.15 | 0.03 |
| Pyruvic acid | 1.18 | 0.60 | 0.74 | 0.22 | 0.92 | 0.61 | 1.92 | 1.43 |
| Succinic acid | 0.72 | 0.25 | 0.47 | 0.12 | **0.84*** | 0.43 | **0.75*** | 0.25 |
| Fumaric acid | 0.06 | 0.02 | **0.04^$^** | 0.01 | 0.06 | 0.02 | **0.06*** | 0.01 |
| Lactic acid | 0.99 | 0.83 | 1.41 | 1.25 | 3.60 | 2.60 | 1.24 | 0.70 |
| Malic acid | 0.36 | 0.04 | **0.26^$^** | 0.05 | **0.34*** | 0.04 | 0.46 | 0.18 |
| 2-oxoglutarate | 0.45 | 0.28 | 0.27 | 0.09 | 0.23 | 0.24 | 0.72 | 1.02 |
| Isocitric acid | 0.04 | 0.00 | 0.04 | 0.00 | 0.04 | 0.00 | 0.04 | 0.00 |
| Citric acid | 0.06 | 0.02 | **0.04^$^** | 0.01 | 0.05 | 0.01 | 0.05 | 0.01 |
| Octanoic acid (C8:0) | 1.18 | 0.32 | 1.08 | 0.37 | 1.34 | 0.31 | 1.45 | 0.51 |
| Nonanoic acid (C9:0) | 1.13 | 0.36 | 1.33 | 0.52 | 1.40 | 0.30 | 1.59 | 0.51 |
| Malonic acid | 0.91 | 0.08 | **0.74^$^** | 0.11 | 0.84 | 0.11 | 0.90 | 0.18 |
| Phenylacetic acid | 0.95 | 0.55 | 0.49 | 0.21 | 0.52 | 0.23 | 0.80 | 0.49 |
| 4-Hydroxyphenylacetic acid | 0.08 | 0.01 | 0.07 | 0.03 | 0.07 | 0.02 | 0.07 | 0.02 |
